# Discover the Power of Lithospermic Acid as Human Carbonic Anhydrase VA and Pancreatic Lipase Inhibitor Through In Silico and In Vitro Studies

**DOI:** 10.1002/ardp.202500046

**Published:** 2025-04-21

**Authors:** Emanuele Liborio Citriniti, Roberta Rocca, Giosuè Costa, Claudia Sciacca, Nunzio Cardullo, Vera Muccilli, Anastasia Karioti, Fabrizio Carta, Claudiu T. Supuran, Stefano Alcaro, Francesco Ortuso

**Affiliations:** ^1^ Dipartimento di Scienze della Salute Università “Magna Græcia” di Catanzaro Catanzaro Italy; ^2^ Net4Science S.r.l. Università “Magna Græcia” di Catanzaro Catanzaro Italy; ^3^ Associazione CRISEA—Centro di Ricerca e Servizi Avanzati per l'Innovazione Rurale Località Condoleo di Belcastro Catanzaro Italy; ^4^ Dipartimento di Scienze Chimiche Università degli Studi di Catania Catania Italy; ^5^ Laboratory of Pharmacognosy, School of Pharmacy Aristotle University of Thessaloniki Thessaloniki Greece; ^6^ NEUROFARBA Department, Sezione di Scienze Farmaceutiche University of Florence Florence Italy

**Keywords:** carbonic anhydrase, docking, lithospermic acid, obesity, pancreatic lipase

## Abstract

Obesity remains a significant global health concern, with limited pharmacological options that balance efficacy and safety. In this study, we identified lithospermic acid (LTS0059529) from *Salvia miltiorrhiza* as a potential dual inhibitor of pancreatic lipase (PL) and human carbonic anhydrase VA (*h*CA VA), two key enzymes in lipid metabolism. Using molecular docking and dynamics simulations, we observed that lithospermic acid interacts with Zn²⁺ in *h*CA VA via its benzofuran carboxylate moiety and forms stable complexes with PL through hydrogen bonding with ASP 205 and π–stacking interactions with PHE 77 and PHE 215. Experimental validation confirmed its inhibitory activity, with *K*_i_ values of 33.1 ± 1.6 μM for PL and 0.69 ± 0.01 μM for *h*CA VA. While its inhibition of *h*CA VA is not isoform‐specific, lithospermic acid demonstrates significant potential as a dual inhibitor, targeting complementary pathways in obesity management. This study is the first to explore its dual action on PL and *h*CA VA, highlighting a promising strategy for future antiobesity therapies. Further research will focus on optimizing selectivity and potency to develop safer and more effective treatments.

## Graphical abstract

Aiming at safe obesity drugs, *Salvia miltiorrhiza* was explored, and lithospermic acid was identified as a dual inhibitor of pancreatic lipase and human carbonic anhydrase VA. In silico and experimental analyses revealed stable binding interactions and good inhibitory effects. Though isoform specificity needs optimization, lithospermic acid shows potential for dual‐action therapies, paving the way for safer obesity treatments.

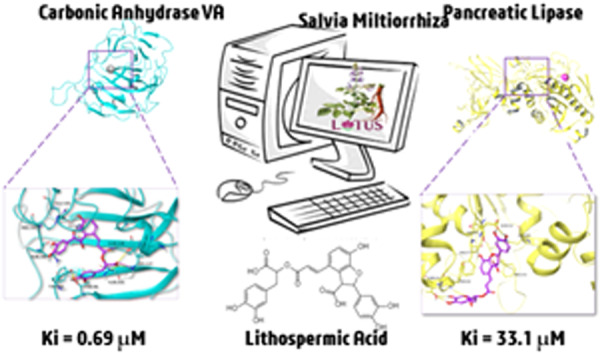

## 1 Introduction

Obesity is a medical condition characterized by the accumulation of excessive fat in the body. It is defined as having a body mass index (BMI) of 30 kg/m² or higher [1]. This is a major health problem worldwide, affecting a large number of people in both developed and developing countries. In the United States alone, it is estimated that two‐thirds of the population struggles with weight issues, with one in every three adults and 20% of adolescents being obese [2]. Obesity can lead to various health issues, such as cardiovascular disease, dyslipidemia, and insulin resistance. These conditions can contribute to diabetes, stroke, gallstones, fatty liver, obesity hypoventilation syndrome, sleep apnea, and certain cancers [3, 4, 5].

Excessive weight gain results from an energy imbalance between intake and expenditure, leading to obesity. The complexity of this condition is influenced by a myriad of factors, including genetics, culture, and society. Obesity has been found to have a high genetic heritability, with several genes associated with adiposity and weight gain. However, other contributing factors include reduced physical activity, sleep problems, endocrine disorders, medications, excessive consumption of carbohydrates and high‐sugar foods, and decreased energy metabolism [6].

It is crucial to recognize that lifestyle and behavioral interventions can only provide moderate efficacy when it comes to treating obesity. To achieve significant results, it is essential to escalate the treatment strategy by incorporating pharmacological and surgical interventions [7]. A significant number of antiobesity drugs have been developed, approved, and subsequently withdrawn within a short period, ranging from a few months to 1 year. This is due to the emergence of serious side effects that were observed in a relatively consistent number of patients immediately after their approval [1, 2]. Due to the lack of safe long‐term options, there is a growing interest in discovering antiobesity drugs. To counter this, researchers have studied numerous targets for potential treatments or preventative measures. Among these targets, pancreatic lipase (PL) and carbonic anhydrase V (*h*CA V) have been identified as significant players in the antiobesity treatment, thanks to their favorable side‐effect profiles [8, 9]. In particular, PL plays a crucial role in breaking down dietary fat by converting triacylglycerols into 2‐monoacylglycerols and free fatty acids. These substances are then absorbed by the body and used in metabolism. By inhibiting this enzyme, it is possible to lower lipid levels and control the amount of fat that enters the bloodstream. Orlistat (ORL) (Xenical) is the only approved medication for treating obesity, but it can cause side effects such as flatulence, fecal incontinence, and steatorrhea [10, 11]. Thus, new enzyme inhibitors are being studied using natural and synthetic libraries of compounds. Some examples of PL inhibitors include bis(sulfonate) derivatives [12], benzothiazole sulfonate derivatives with azomethine [13], and Pd(II)‐Schiff base complexes [14], and analogs of natural products as isoflavones [15] and neolignans [16].

On the other hand, the *h*CA VA/B isoforms are mitochondrial metalloenzymes involved in metabolic processes such as de novo lipogenesis and fatty acid biosynthesis. In humans, *h*CA VA is primarily located in the liver, skeletal muscle, and kidneys. Conversely, *h*CA VB is present in several tissues, including the pancreas, kidneys, salivary glands, spinal cord, heart, and skeletal muscle but not in the liver. Both isoforms are present in adipocytes, astrocytes, and neuronal cells [17, 18]. It is a well‐established fact that some CA VA/B inhibitors possess a broad‐spectrum inhibition of metabolism, while others have significant effects on specific metabolic pathways. Among these, pyruvate metabolism is the most dramatically affected by CA inhibition, followed by fatty acid metabolism, and, finally, succinate metabolism. These findings provide conclusive evidence of the role of mitochondrial CAs in metabolism and fatty acid biosynthesis [19]. Inhibition of these enzymes to develop antiobesity medication was not considered until the 2000s when Solvay Pharmaceuticals—which no longer exists—and some academic research groups initiated a program to obtain carbonic anhydrase inhibitors (CAIs) with antiobesity properties [20].

Research on antiobesity CAIs has been limited. Currently, there are three main approaches to this study, which have led to many interesting advancements over the past two decades. These approaches include: (i) repurposing drugs initially discovered for other uses, (ii) screening natural products or synthetic libraries, and (iii) conducting de novo drug design studies based on previously identified leads or structural biology data [9]. Considering the progress made so far, the search for new promising compounds in antiobesity therapy is crucial in the fight against this disease and an exciting opportunity for discoveries.

Traditional “one target–one drug” approaches are shifting toward multi‐target strategies, particularly for complex diseases like metabolic syndrome, with polypharmacology emerging as a more effective approach to enhancing efficacy and minimizing side effects. Recent studies highlight that multi‐target therapies outperform single‐target approaches in both efficacy and patient outcomes [21]. In obesity treatment, simultaneously targeting PL and *h*CA VA offers a unique synergistic mechanism by reducing fat absorption while modulating lipid metabolism. In this study, we investigate for the first time the dual inhibition of PL and *h*CA VA, introducing a novel multi‐target strategy that may enhance metabolic regulation and optimize therapeutic efficacy in obesity treatment.

Natural products from traditional medicinal plants and microbial sources offer a valuable pool of novel drug leads. Among these sources, traditional Chinese medicinal herbs stand out as a rich and promising reservoir of lead compounds. With their potential to treat various pathological conditions, including obesity, these herbs could hold the key to developing effective treatments for a wide range of illnesses [22, 23, 24, 25]. Among these plants, *Salvia miltiorrhiza* is an extensively used traditional Chinese medicine, known as Danshen [26], to treat cardiovascular disease, hepatic injury, and other health problems in Asian countries [27]. Over the past few decades, researchers have studied its pharmacological effects and have identified multiple bioactive compounds, such as phenolic acids, tanshinone, related quinone derivatives, and protocatechualdehyde [26, 28]. Recent studies suggest that *S. miltiorrhiza* extract has the potential to combat obesity in rats that have been fed high‐fat diets, although the underlying targets are not well defined [29]. Accordingly, we conducted a comprehensive virtual screening of a compound database extracted from *S. miltiorrhiza*, with the main goal of thoroughly investigating their interactions with both *h*CA V and PL.

## 2 Results and Discussion

### 2.1 Computational Studies

**Figure 1:**
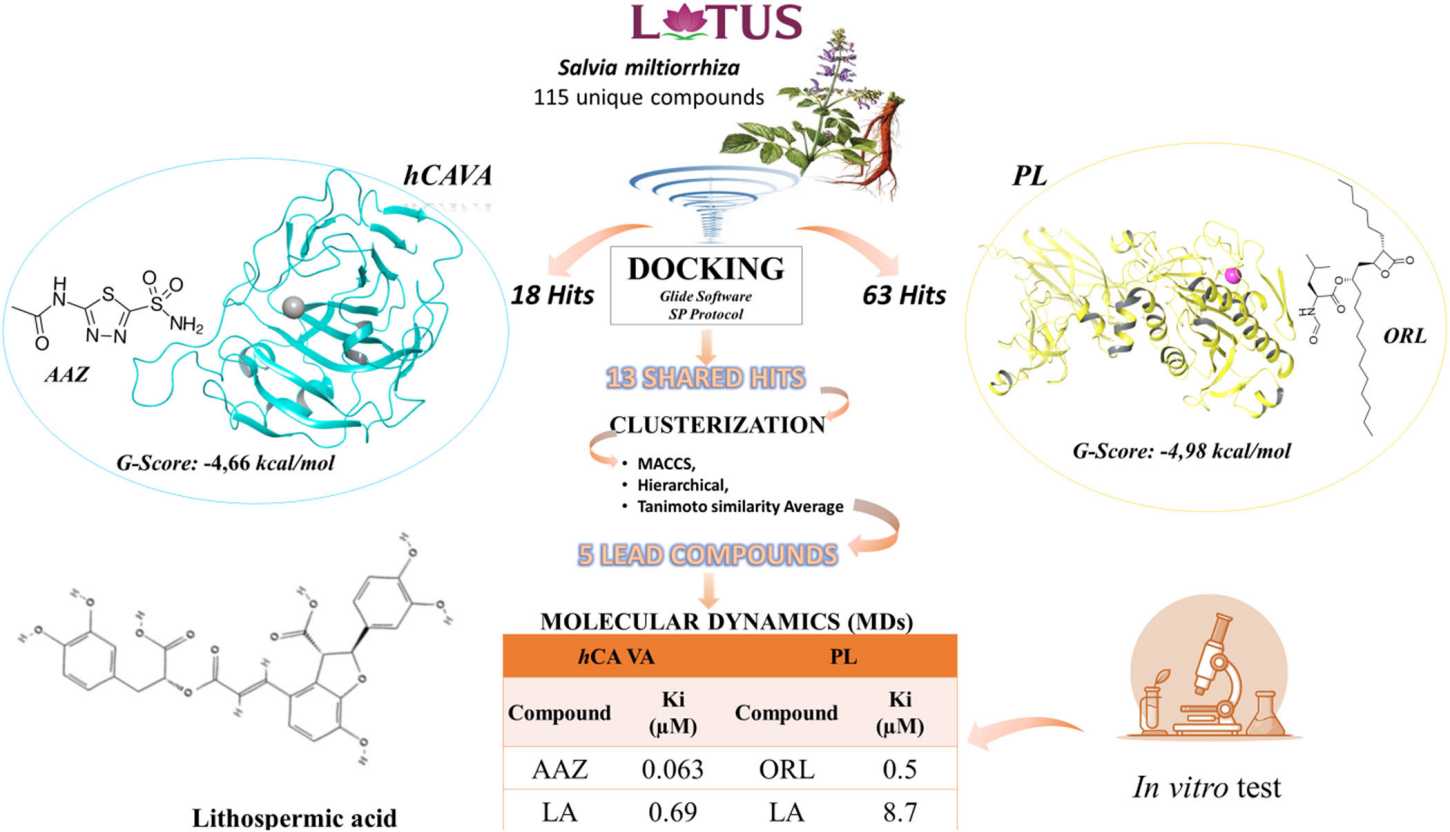
Workflow applied in this study.

Our ongoing research aims to uncover novel natural chemical compounds with antiobesity properties. In Figure 1, we offer a comprehensive overview of the employed process, which contributes to the enhanced clarity and coherence of the approach.

Our approach involves screening the chemical database of *S. miltiorrhiza*, which yielded 115 compounds. Thus, we prepared all conceivable stereoisomers and protonation states at pH 7.4, leading to a comprehensive database of 504 chemical structures.

To evaluate the precision and reliability of our docking procedure, we initially conducted Glide SP docking calculations for two well‐known active compounds, acetazolamide (AAZ) and ORL, against *h*CA VA and PL, respectively. We utilized the Glide Score of their best docking pose as a *cut‐off* to screen the Salvia database. Specifically, we considered the threshold values of –4.66 and –4.98 kcal/mol for *h*CA VA and PL, respectively. This procedure led us to identify 18 and 63 potential inhibitors of *h*CA VA and PL, respectively. Out of these, we found 13 shared compounds with similar chemical structures and clustered them to obtain five promising *hits*, which showed good potential (Table 1).

**Table 1:** The five best *hits*, proposed as potential *h*CA VA/PL inhibitors. For each *hit*, the following information is provided: Lotus ID Database, Common Name, 2D structure, Glide Scores values (kcal/mol) for *h*CA VA and PL.

Lotus ID database	Common name	2D structure	Glide score PL (kcal/mol)	Glide score *h*CA VA (kcal/mol)
LTS0059529	Lithospermic acid	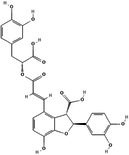	–6.13	–5.24
LTS0145253	—	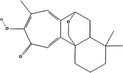	–5.37	–4.97
LTS0029118	2α‐Acetoxysugiol	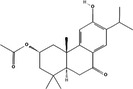	–5.29	–4.78
LTS0153650	—	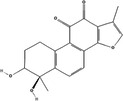	–7.66	–4.90
LTS0121786	Danshinspiroketallactone	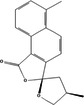	–8.44	–4.78

For each hit, we analyzed the docking poses of the stereoisomer with the best Glide score for both targets. All compounds exhibited good Glide score against PL, with values ranging between –5.29 and –8.44 kcal/mol. Conversely, for the *h*CA VA, the lithospermic acid (LTS0059529) obtained the best Glide score value of –5.24 kcal/mol, while the other compounds exhibited Glide score values ranging from –4.78 to –4.97 kcal/mol. Upon analysis of chemical structures, we have predominantly observed partly aromatic polycyclic structures and only one polyphenol compound containing a characteristic 2,3‐dihydro‐7‐hydroxy‐3‐benzofurancarboxylic acid core. In particular, the polycyclic moiety of these compounds exhibits different combinations of both aromatic and nonaromatic portions, leading to different scaffolds.

The analysis of their binding mode disclosed the hydrogen bonds (H‐bonds) as the most important intermolecular interactions in both complexes for all obtained hits (Supporting Information S2: Figure S1–2), except for LTS0029118, which established a π–π interaction with residue PHE 77 when complexed with lipase (Supporting Information S2: Figure S1H–L and S2H–L). A detailed overview of how the compounds interact with both targets is included in the Supplementary Information (Section “Analysis of docking binding poses”).

Molecular Dynamics simulations (MDs) were conducted to assess the stability of the binding mode in both *h*CA VA and PL for the five promising hits selected with the SBVS. The main objective of conducting MDs is to provide critical insights into the compounds’ interactions with the target proteins and facilitate the identification of the most suitable candidates for experimental investigations. In both examined targets, the simulations showed an unstable binding mode for the two compounds with the code LTS0029118 and LTS0153650, indicating a tendency for them to leave the binding site (Supporting Information S2: Figure S3). This observation explains the lack of stable interactions occurring in more than 30.0% of the 200 ns simulation time, as shown in Supporting Information S2: Figure S5B,C,F,G. Despite having a stable binding mode to the PL, the compound LTS0121786 is highly unstable in the *h*CA VA binding site, where it fails to maintain crucial interactions (Supporting Information S2: Figures S3 and S4D–H). During the simulation, the compound LTS0121786 maintained a π–π interaction with HIS 263 in the PL binding site for 33% of the time, consistent with the docking pose. Conversely, the H‐bond observed in the docking pose with SER152 (Supporting Information S2: Figure S1–2L) was replaced by a stable H‐bond with PHE 77 (Supporting Information S2: Figure S4H).

The root means square deviation (RMSd) trend of LTS0145253 was found to be higher compared with the reference compounds AAZ and ORL. This observation was especially evident in the *h*CA VA binding site, as shown in Supporting Information S2: Figure S3. The tropolone ring was involved in different interactions compared with the docking poses of both targets (Supporting Information S2: Figure S1–2B, S1–2G). Specifically, in the *h*CA VA, the hydroxyl group of the ligand established a stable H‐bond acceptor with the LYS 127 and a water bridge between the GLU 105 and the carbonyl moiety (Supporting Information S2: Figure S4A). Conversely, in the PL binding site, we observed an H‐bond acceptor between the hydroxyl group of LTS0145253 and the ARG 23, occurring for 30.0% of the simulation time (Supporting Information S2: Figure S4E) and two water bridges with TYR 114 and THR 115.

**Figure 2:**
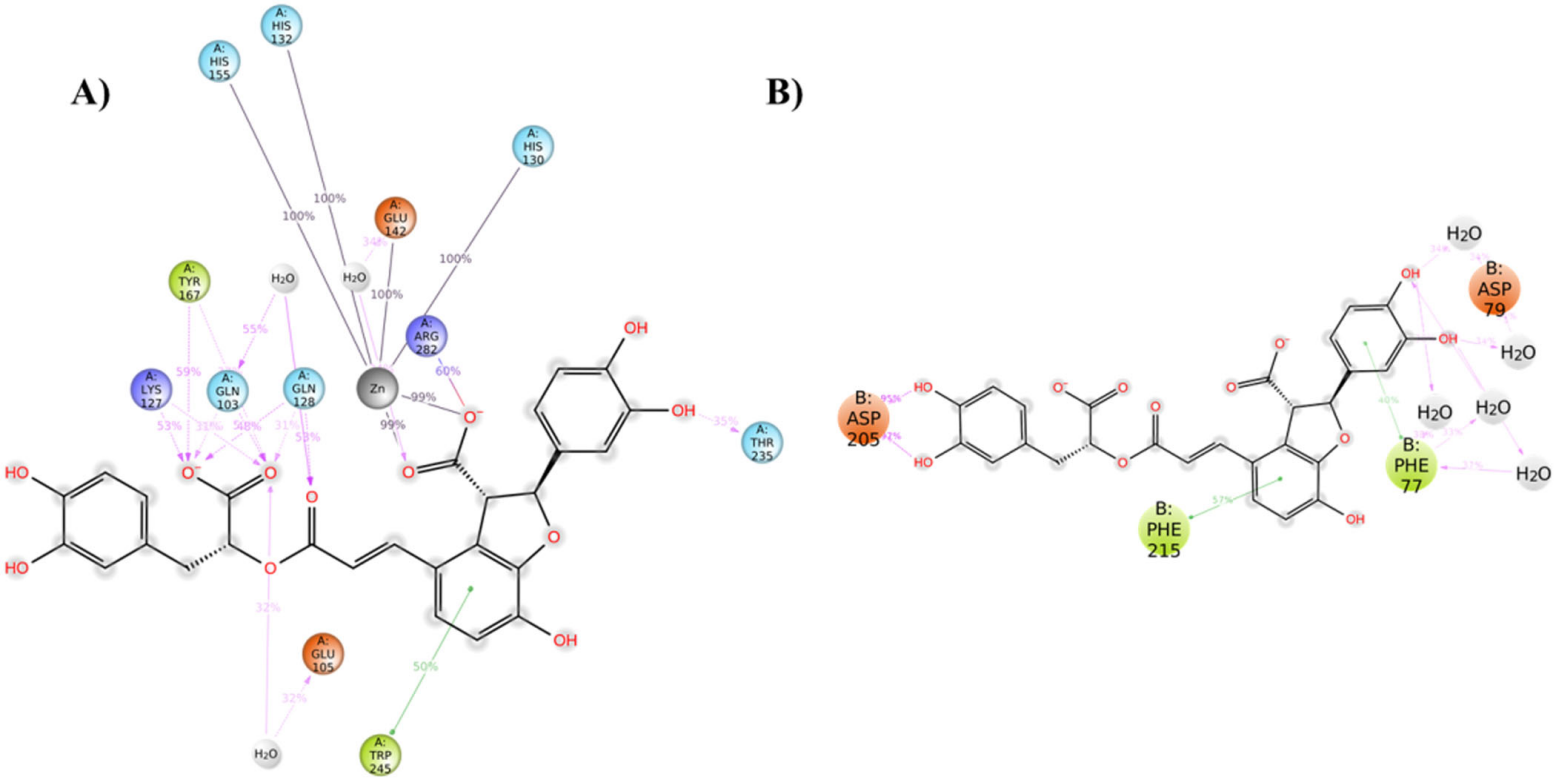
Ligand atom interactions for the compound LTS0059529 with the residues of (A) *h*CA VA and (B) PL. Only interactions that occur more than 30.0% of the simulation time in 200 ns of the trajectory are shown.

LTS0059529 is the only compound with comparable stability of its binding mode in both *h*CA VA and PL, resulting in a similar or even better behavior than the reference compounds (Supporting Information S2: Figure S3). Initially, the ligand adjusts its conformation to achieve a better fit both *h*CA VA and PL pockets, which helps maintain a stable binding mode. Interestingly, during the entire simulation, the LTS0059529 is the only compound able to interact with the Zn^2+^ ion of the *h*CA VA through the carboxyl group of its benzofuran ring (Figure 2A). Moreover, several interactions, occurring more than 30.0% of the simulation time, were established: (a) a H‐bond donor with the THR 235; (b) several H‐bonds acceptors with GLN 128, LYS 127 and GLN 103; (c) a π–π interaction with TRP245; (d) two water bridges with GLN 103 and GLU 105; (e) a salt bridge with the ARG 282 (Figure 2A).

Regarding the LTS0059529 complex with the PL, we observed two H‐bonds between the pyrocathecol of the ligand and the residue ASP 205. Additionally, two π–π interactions were established between the benzofuran ring and the linked pyrocatecol with the PHE 215 and PHE 77, respectively. Finally, several water bridges were established among the pyrocatecol ring linked to the benzofuran ring and the residues ASP 79 and PHE 77 (Supporting Information S2: Figure 2B).

These data suggest that the LTS0059529 compound is the most effective in recognizing both targets, making it a promising candidate for further exploration and experimental testing. Furthermore, the other compounds have unfortunately not been commercially available. To ensure the reliability of our MD simulations for the LTS0059529 compound, we performed triplicate MD runs (Supporting Information S2: Figure S5–6). The analysis of the RMSD trend indicates that the LTS0059519 compound, when complexed with hCA VA, exhibits relatively stable RMSD values across all three molecular dynamics (MD) runs. Specifically, md Run 1, md Run 2, and md Run 3 consistently average around 4 Å, which suggests stable binding behavior (Figure S5A). Similarly, in the LP complex, both md run 1 and md run 2 display RMSd values fluctuating around 4 Å throughout the simulation. However, md run 3 exhibits a spike in RMSd values above 10 Å between 40 and 90 ns, before stabilizing and maintaining a consistent overall binding mode (Supporting Information S2: Figure S5B). Furthermore, the analysis of ligand–atom interactions reveals a consistent pattern of key residues involved in the binding across all three MD runs, confirming a stable binding mode throughout (Supporting Information S2: Figure 2–S6).

### 2.2 Biophysical Assays

The inhibition of PL (from porcine pancreas; see Supporting Information) was assessed using previously described spectrophotometric methods [15, 16, 30]. The inhibitory activity was determined as the concentration required to inhibit 50% of the enzyme activity (IC_50_; µM). Thus, a lower value indicates higher inhibitory activity. The antiobesity drug ORL (IC_50_: 0.4 ± 0.05 µM) was used as a positive control, and the result aligned with the value reported in the literature [31]. LTS0059529 exhibited promising PL inhibitory activity with an IC_50_ value of 5.4 ± 0.17 µM (Table 2).

**Table 2:** Calculated IC_50_, *K*_*i*_ and inhibition mode of LTS0059529 towards PL.

ID	IC_50_ (µM) ± SD	*K*_i_ (µM)	Inhibition mode
LTS0059529	5.45 ± 0.17	33.1 ± 1.6	Competitive
ORL	0.416 ± 0.02	0.528 [31]	Competitive

**Figure 3:**
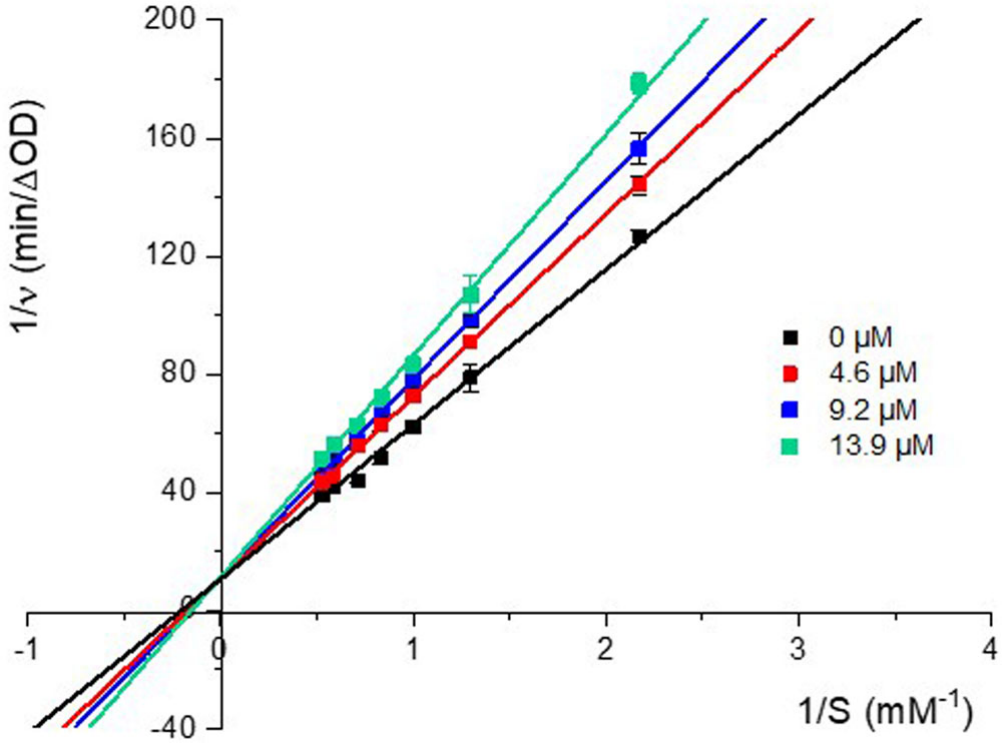
Lineweaver–Burk plot of PL inhibition in the presence of LTS0059529 (from 0 to 13.9 µM). ν is the initial velocity of the reactions. S represents the concentration of *p*‐nitrophenol released by reaction of PL with *p*‐nitrophenyl butyrate.

The inhibition of PL by LTS0059529 on PL was also determined using Lineweaver–Burk graphs, which plot the reciprocal of initial velocity (*v*_*0*_) against the reciprocal of substrate concentration. The LTS0059529 inhibits PL activity competitively, as illustrated in Figure 3, as the L–B plot results in data lines crossed on the y‐axis. As a competitive inhibitor, LTS0059529 does not affect the *v*_max_ of the reaction (0.091 ± 0.002 mM min^−1^). A *K*_i_ value of 33.1 ± 1.6 µM for the enzyme‐inhibitor complex was determined by plotting the slope of L–B plot lines versus the inhibitor concentration (Supporting Information S2: Figure S7–8). Using the same procedure outlined in this study, ORL was shown to be a competitive inhibitor, with a *K*_i_ value of 0.528 µM [32].

Although LTS0059529 is not as potent an inhibitor as ORL, it is important to note that ORL itself is derived from chemically modified polyketides, which act as PL inhibitors. In this context, LTS0059529 can be regarded as a natural scaffold for the development of a new class of synthetic lipase inhibitors.

In vitro inhibition profiles of LTS0059529 and the reference drug AAZ on the catalytically active *h*CA isoforms I, II, VA, VB, IX, and XII were determined through the stopped‐flow CO_2_ hydrase assay [33] and reported in Table 3.

**Table 3:** Inhibition data of LTS0059529 and the reference drugs acetazolamide (AAZ) on *h*CA isoforms I, II, VA, VB, IX, and XII by the stopped flow CO_2_ hydrase assay [33].

*K*_*i*_ (μM)^a^						
Compound	***h*** **CA I**	***h*** **CA II**	***h*** **CA VA**	***h*** **CA VB**	***h*** **CA IX**	***h*** **CA XII**
LTS0059529	> 100	> 100	0.69 ± 0.01	0.60 ± 0.02	0.31 ± 0.01	0.0048 ± 0.003
AAZ	0.25 [33]	0.012 [33]	0.074 ± 0.001	0.063 ± 0.002	0.054 ± 0.002	0.0025 [33]

a Mean from 3 different assays, by the stopped flow technique (errors were in the range of ± 5%–10% of the reported values).

As general consideration on data in Table 3, the natural product LTS0059529 resulted in an ineffective inhibitor on the abundantly expressed *h*CAs I and II with *K*_i_ values > 100 μM. Conversely, all the remaining isoforms were inhibited with *K*_i_ potencies spanning between 0.0048 and 0.69 μM.

Specifically, the mitochondrial expressed *h*CAs VA and VB showed *K*_i_ inhibition values of 0.69 and 0.60 μM, respectively, thus, with the latter being very slightly (i.e., 1.15‐fold) more potently inhibited. Quite interestingly, the same inhibition ratio of the LTS0059529 was maintained from the reference AAZ (i.e., 1.17‐fold), thus suggesting that both compounds, although highly different, did not contain structural features able to discriminate between the two isoforms sharing a homology sequence of 59.3% [34].

The LTS0059529 showed a twice more potent inhibition potency for the tumor‐associated *h*CA IX isoform (i.e., *K*_i_ of 0.31 μM) when compared with the mitochondrial ones VA/B. Even more, the second tumor‐associated isoform *h*CA XII was strongly inhibited by LTS0059529 with a *K*_i_ value of 4.8 nM [34].

## 3 Conclusion

The discovery of effective, safe drugs for obesity remains a significant challenge, with few options available that combine efficacy with minimal side effects. Several antiobesity drugs have been withdrawn from the market because adverse effects became apparent after their approval [1, 2, 35]. Natural products continue to be a rich source of bioactive agents, offering structural diversity that complements synthetic compounds [36]. However, efficiently navigating this diverse chemical space remains a hurdle for medicinal chemists and pharmacologists. Virtual screening has shown significant promise in drug discovery and is crucial for identifying active lead compounds from natural products.

Our study represents a significant advancement in understanding the antiobesity effects of *S. miltiorrhiza* by identifying its bioactive molecules using in silico methods [29]. While previous research has demonstrated that *S. miltiorrhiza* extracts reduce body weight, triglycerides, and cholesterol levels in diet‐induced obese mice—effects linked to lipid metabolism modulation, gut microbiota composition, and the regulation of adipogenesis and lipolysis‐related genes—these studies have primarily focused on crude extracts, leaving the specific active compounds unidentified [37]. In this study, we provide a novel contribution by identifying lithospermic acid (LTS0059529) as a potent inhibitor of two key obesity‐related targets: PL and human carbonic anhydrase VA (*h*CA VA). In silico analyses revealed lithospermic acid's interaction with Zn^2+^ in the active site of *h*CA VA through the carboxylate moiety of its benzofuran ring, and strong stability in complexes with PL due to hydrogen bonding with ASP 205 and π‐stacking interactions with PHE 77 and PHE 215. Notably, PHE 77 has been recognized as crucial in PL binding with natural molecules [38, 39].

Further experimental validation confirmed that lithospermic acid inhibits both phospholipase (PL) and human carbonic anhydrase VA (*h*CA VA), indicating its potential to regulate these enzymes. Although lithospermic acid's inhibition of *h*CA VA is not specific to any particular isoform, these findings suggest that it could serve as a dual inhibitor. This finding highlights the need for future studies to optimize its selectivity and enhance its biological activity, specifically targeting the VA isoform of *h*CA as a promising avenue for developing potent antiobesity compounds.

Our study is the first to explore the potential dual inhibitors of PL and human carbonic anhydrase VA (*h*CA VA), highlighting their synergistic action against obesity. The findings suggest that lithospermic acid offers a promising strategy for targeting these two key molecular pathways. This study paves the way for further studies to optimize and refine dual inhibition approaches, potentially leading to more effective and safe treatments for obesity.

## 4 Experimental

### 4.1 Molecular Modeling

#### 4.1.1 Database Preparation

The chemical components of the *S. miltiorrhiza* species were downloaded from the Lotus database (https://lotus.naturalproducts.net/), obtaining 191 compounds [40, 41]. After removing the duplicates, 115 unique compounds were prepared using Ligprep at pH 7.4, with tautomers and stereoisomers explicitly considered [42]. Thus, we obtained a final database of 504 structures to undergo docking simulations.

#### 4.1.2 Receptor Preparation

The Uniprot Database lacks 3D structures for the *h*CA VA, while four experimental models are available for the PL [43, 44] (https://www.uniprot.org/). From the Protein Data Bank website [45, 46] (https://www.rcsb.org/), we selected and obtained the crystallographic structure with the PDB code 1LPB as a PL 3D model [47]. This model, which has been widely used in several previous works, exhibits the best resolution and sequence completeness, making it an ideal choice for this study [16, 48, 49, 50]. Conversely, we utilized a homology model that was obtained and utilized in previous work to study *h*CA VA [51]. This model served as a valuable tool in our investigation of *h*CA VA and its inhibitors. Both structures were prepared with the Protein Preparation Wizard tool [52], considering OPLS 2005 as a force field [53]. Thus, the correct bond orders were assigned, and missing atoms, side chains, and loops were built. Since lipase is cocrystallized with colipase in the presence of methoxy undecyl phosphonic acid (MUP) as an inhibitor and β‐octylglucoside (BOG) as a surfactant, these molecules were also removed.

#### 4.1.3 Receptor Validation

For the *h*CA VA, the accuracy and reliability of our protocol were previously validated in a published study [51]. This validation involved rigorous enrichment experiments confirming the robustness of our docking approach for *h*CA VA. For the current study, we have applied the same validated protocol for PL to ensure the reliability of our results. Specifically, for the target protein of PL, we performed enrichment experiments with 225 known inhibitors (Supporting Information S2: Table S1), extrapolated from the BindingDB database [54], and 4910 decoys obtained using the DUDE database [55]. By leveraging the protocol, which has proven effective for *h*AC VA, we ensure that our docking results for PL are equally reliable with an AUC of 0.67 (Supporting Information S2: Figure S7). This consistent application of a validated protocol provides good support for the accuracy and robustness of our findings.

#### 4.1.4 Docking Simulations

Docking calculations were performed using the optimized structures for PL and *h*CA VA with the help of Glide software [56]. For *h*CA VA, the rigid receptor grid was centered on the co‐crystallized ligand. For PL, the grid center was defined as the centroid of the residues Asp79, Ser152, and His263, which form the catalytic triad essential for the enzyme's activity. This choice was made due to the absence of a co‐crystallized inhibitor to directly define the active site. The dimension of the inner box of the grid was set to 15 × 15 × 15 Å. Thus, the Glide SP protocol was applied to generate 10 poses per ligand, using the default parameters, as validated in our previous work [51]. The docking protocol was applied to AAZ and ORL, two known inhibitors of *h*CA VA and PL, respectively, to establish a cut‐off value for selecting potential inhibitors. The shared *hits* were clustered using the K‐means algorithm, based on MACCS keys [57] to describe the compounds and the Tanimoto similarity as the comparison metric [58]. The optimal number of clusters was determined using the elbow method, ensuring meaningful groupings of similar compounds. For each cluster, the most representative compound was selected as the closest to the cluster centroid in terms of Tanimoto similarity, as it best captured the cluster's characteristics. We identified only five clusters, each containing a single compound, except for the first cluster, which is the most populated with nine compounds. Among these, LTS0059529 was determined as the centroid of the first cluster.

The outcome of this process allows the identification and selection of the most effective potential inhibitors for further analysis.

#### 4.1.5 MDs

The complexes of both studied targets with the best *hits* selected from virtual screening were submitted to 200 ns of MDs using Desmond ver. 4.4 [59]. All the molecular systems under study were placed inside an orthorhombic box with a thickness of 10 Å, and then immersed in a solvent using the TIP3P [60] water model parameters. 5 Na^+^ counterions were added to neutralize the charge of the systems. The solvated model was further optimized and then relaxed using the Martyna–Tobias_Klein isobaric‐isothermal ensemble (MTK_NPT). The systems were then equilibrated using the NVT ensemble at 10 K and the NPT ensemble at 300 K and 1 atm with the Berendsen thermostat‐barostat. The trajectory frames of the systems were sampled every 200 ps and analyzed using the Simulation Interaction Diagram and the Simulation Event Analysis. These analyses enabled a detailed investigation of the geometrical and thermodynamic properties of the trajectories that were obtained. Finally, the same protocol was applied to both the unbounded targets and their complexes with the known inhibitors, previously used in the docking process. This method ensdured reliable and consistent results across all studied systems.

### 4.2 Isolation of LTS0059529 (Lithospermic Acid)

The compound LTS0059529 corresponds to the lithospermic acid, which was isolated from *S. miltiorrhiza* as reported from some of us and is ≥ 98% pure by HPLC [34].

### 4.3 PL Inhibition

#### 4.3.1 Measurements of Lipase Inhibition

The inhibition of PL (EC 3.1.1.3; triacylglycerol acyl hydrolase; PL) from porcine pancreas (Type II, ≥ 125 units/mg protein) was performed using the conditions previously reported [15, 16]. In a 96‐well microplate, the PL solution (5 mg/mL in phosphate buffer; 15 μL), and different aliquots (2, 4, 6, and 10 μL) of LTS0059529 (stock solutions 0.14 mM and 0.20 mM in MeOH) or ORL used as positive control (6.7 μM in phosphate buffer) were mixed. The reactions were incubated at 37°C for 10 min. Then, the substrate *p*‐nitrophenyl butyrate (3.2 mM in H_2_O/DMF 70:30, 10 μL) was pipetted, and the microplate was incubated at 37°C for 30 min under moderate shaking. The plate measurements were performed at 405 nm with an Agilent BioTek Synergy H1 Multimode Reader equipped with the Gen5 software. The amount of methanol used in the experiment did not affect the lipase inhibitory activity. The following equation gave the inhibition percentage of enzyme activity and was employed to elaborate the data:

(1) $$\%\mathrm{inhibition}=\frac{{\text{OD}}_{\text{control}}-{\text{OD}}_{\text{sample}}}{{\text{OD}}_{\text{control}}}\times 100,$$

where OD_control_ represents the measured optical density for the enzyme–substrate mixture, and OD_sample_ represents the optical density of the enzyme–substrate mixture in the presence of the inhibitor. The inhibition data were analyzed as a dose–response curve, and the concentration required to inhibit the 50% activity of the enzyme (IC_50_) was determined.

#### 4.3.2 Kinetics of PL Inhibition

The mode of inhibition of PL in the presence of LTS0059529 was determined by elaboration of UV‐vis spectroscopic data with Lineweaver–Burk plots [15, 16]. The experiments were performed in 96‐well plates employing the enzyme (120 U/mL in phosphate buffer; 10 μL), the LTS0059529 (at selected concentrations chosen based on the IC_50_ value), and p‐nitrophenyl butyrate (from 0.3 to 1.9 mM) in a final volume of 200 μL. The absorbance was read at 405 nm every 1 min for 30 min at 37°C. The competitive inhibition constants *K*_i_ was calculated from the following equation.

(2) $$v_{0}=\frac{v_{\max}S}{K_{m}\left(1+\frac{I}{Ki}\right)+S},$$

where *ν*_0_ is the initial velocity in the absence and presence of the inhibitor, *S* and *I* are the concentrations of substrate and inhibitor, respectively; *ν*_max_ is the maximum velocity, *K*_m_ is the Michaelis–Menten constant. The replot of slopes (*K*_m_/*ν*_max_) from Lineweaver–Burk plot versus the inhibitor concentration afforded *K*_i_ value as well.

### 4.4 Carbonic Anhydrase Inhibition Assays

An Applied Photophysics stopped‐flow instrument was used to assay the CA‐catalyzed CO_2_ hydration activity [33]. Phenol red (at a concentration of 0.2 mM) was used as an indicator, working at the absorbance maximum of 557 nm, with 20 mM Hepes (pH 7.4) as a buffer, and 20 mM Na_2_SO_4_ (to maintain constant ionic strength), following the initial rates of the CA‐catalyzed CO_2_ hydration reaction for a period of 10 − 100 s. The CO_2_ concentrations ranged from 1.7 to 17 mM for the determination of the kinetic parameters and inhibition constants. Enzyme concentrations ranged between 5 and 12 nM. For each inhibitor, at least six traces of the initial 5%−10% of the reaction were used to determine the initial velocity. The uncatalyzed rates were determined in the same manner and subtracted from the total observed rates. Stock solutions of the inhibitor (0.1 mM) were prepared in distilled−deionized water, and dilutions up to 0.01 nM were done thereafter with the assay buffer. Inhibitor and enzyme solutions were preincubated together for 15 min at room temperature before the assay, to allow for the formation of the E − I complex. The inhibition constants were obtained by nonlinear least‐squares methods using PRISM 3 and the Cheng–Prusoff equation as reported earlier and represent the mean from at least three different determinations. All CA isoforms were recombinant proteins obtained in house, as reported earlier [34, 61].

## Conflicts of Interest

The authors declare no conflicts of interest.

## Supporting information

archpharm supplmat inchi.

Supporting information.

## Data Availability

The data that support the findings of this study are available on request from the corresponding author. The data are not publicly available due to privacy or ethical restrictions.
