# Supplementary material for: Discover the Power of Lithospermic Acid as Human Carbonic Anhydrase VA and Pancreatic Lipase Inhibitor Through In Silico and In Vitro Studies
Source: Arch Pharm (Weinheim). 2025 Apr 21;358(4):e3128. doi: 10.1002/ardp.202500046 (PMC12010950; doi:10.1002/ardp.202500046)
Supplement: Supplementary file 1 — archpharm supplmat inchi. [file ARDP-358-e3128-s001.doc]

**Supplemental Material: Novel Compounds and Biological Screening Results**

Discover the Power of Lithospermic Acid as human Carbonic Anhydrase VA and Pancreatic Lipase Inhibitor through In Silico and In Vitro Studies

Emanuele Liborio Citriniti,^1‡^ Roberta Rocca,^1,2,3‡^ Giosuè Costa,^1,2^ Claudia Sciacca,^4^ Nunzio Cardullo,^4^ Vera Muccilli,^4^ Anastasia Karioti,^5^ Fabrizio Carta,^6^ Claudiu T. Supuran,^6^ Stefano Alcaro,^1,2,3*^ Francesco Ortuso,^1,2^

1 Dipartimento di Scienze della Salute, Università “Magna Græcia” di Catanzaro, Viale Europa, 88100 Catanzaro, Italy

2 Net4Science S.r.l., Università “Magna Græcia” di Catanzaro, Viale Europa, 88100 Catanzaro, Italy

3 Associazione CRISEA—Centro di Ricerca e Servizi Avanzati per l’Innovazione Rurale, Località Condoleo di Belcastro, 88055 Catanzaro, Italy

4 Dipartimento di Scienze Chimiche, Università degli Studi di Catania. V.le A. Doria 6, 95125 Catania

5 Laboratory of Pharmacognosy, School of Pharmacy, Aristotle University of Thessaloniki, University Campus, 54124 Thessaloniki, Greece

6 NEUROFARBA Department, Sezione di Scienze Farmaceutiche, University of Florence, Via Ugo Schiff 6, 50019 Florence, Italy

*Correspondence:

Prof. Stefano Alcaro, Dipartimento di Scienze della Salute, Università “Magna Græcia” di Catanzaro, Viale Europa, 88100 Catanzaro, Italy

Email: alcaro@unicz.it

^‡^These authors contributed equally

|  |  | **KI (µM)*** | | | | | | |
| --- | --- | --- | --- | --- | --- | --- | --- | --- |
| ***Hits*** | **InChI codes** | ***h*CA I** | ***h*CA II** | ***h*CA VA** | ***h*CA VII** | ***h*CA IX** | ***h*CA XII** | **Lipase pancreatic** |
| **Lithospermic acid** | InChI=1S/C27H22O12/c28-15-5-1-12(9-18(15)31)10-20(26(34)35)38-21(33)8-4-13-2-7-17(30)25-22(13)23(27(36)37)24(39-25)14-3-6-16(29)19(32)11-14/h1-9,11,20,23-24,28-32H,10H2,(H,34,35)(H,36,37)/b8-4+/t20-,23+,24-/m1/s1 | >100 | >100 | 0.69 ± 0.01 | 0.60 ± 0.02 | 0.31 ± 0.01 | 0.0048 ± 0.003 | 33.1 ± 1.6 |
