# Supplementary material for: Discover the Power of Lithospermic Acid as Human Carbonic Anhydrase VA and Pancreatic Lipase Inhibitor Through In Silico and In Vitro Studies
Source: Arch Pharm (Weinheim). 2025 Apr 21;358(4):e3128. doi: 10.1002/ardp.202500046 (PMC12010950; doi:10.1002/ardp.202500046)
Supplement: Supplementary file 2 — Supporting information. [file ARDP-358-e3128-s002.docx]

**Supporting Information**

Discover the Power of Lithospermic Acid as human Carbonic Anhy-drase VA and Pancreatic Lipase Inhibitor through In Silico and In Vitro Studies

Emanuele Liborio Citriniti,^1‡^ Roberta Rocca,^1,2,3‡^ Giosuè Costa,^1,2^ Claudia Sciacca,^4^ Nunzio Cardullo,^4^ Vera Muccilli,^4^ Anastasia Karioti,^5^ Fabrizio Carta,^6^ Claudiu T. Supuran,^6^ Stefano Alcaro,^1,2,3*^ Francesco Ortuso,^1,2^

1 Dipartimento di Scienze della Salute, Università “Magna Græcia” di Catanzaro, Viale Europa, 88100 Catanzaro, Italy

2 Net4Science S.r.l., Università “Magna Græcia” di Catanzaro, Viale Europa, 88100 Catanzaro, Italy

3 Associazione CRISEA—Centro di Ricerca e Servizi Avanzati per l’Innovazione Rurale, Località Condoleo di Belcastro, 88055 Catanzaro, Italy

4 Dipartimento di Scienze Chimiche, Università degli Studi di Catania. V.le A. Doria 6, 95125 Catania

5 Laboratory of Pharmacognosy, School of Pharmacy, Aristotle University of Thessaloniki, University Campus, 54124 Thessaloniki, Greece

6 NEUROFARBA Department, Sezione di Scienze Farmaceutiche, University of Florence, Via Ugo Schiff 6, 50019 Florence, Italy

*Correspondence:

Prof. Stefano Alcaro, Dipartimento di Scienze della Salute, Università “Magna Græcia” di Catanzaro, Viale Europa, 88100 Catanzaro, Italy

Email: alcaro@unicz.it

**Contents**

**FIGURE S1. A-E)** 2D representation of *h*CA VA complexed with **A)** LTS0059529, **B)** LTS0145253, **C)** LTS0029118, **D)** LTS0153650, **E)** LTS0121786. **F-L)** 2D representation of PL complexed with **F)** LTS0059529, **G)** LTS0145253, **H)** LTS0029118, **I)** LTS0153650, **L)** LTS0121786. H-bonds, salt bridges and stacking interactions are shown as magenta, red-blue and green lines, respectively.

**FIGURE S2: A-E)** Three-dimensional representation of *h*CA VA complexed with **A)** LTS0059529, **B)** LTS0145253, **C)** LTS0029118, **D)** LTS0153650, **E)** LTS0121786. **F-L)** Three-dimensional representation of PL complexed with **F)** LTS0059529, **G)** LTS0145253, **H)** LTS0029118, **I)** LTS0153650, **L)** LTS0121786. *h*CA VA and PL are depicted as cyan and yellow cartoons, respectively. Ligands are depicted as sticks, while the amino acid residues involved in the most relevant contacts with ligands as cyan and yellow sticks. The zinc ion of the *h*CA VA is depicted as a grey sphere. H-bonds, salt bridges and stacking interactions are shown as yellow, magenta and cyan dash lines, respectively.

**Analysis of docking binding poses**

**FIGURE S3** RMSD trends of LTS0059519 (green line), LTS0145253 (blue line), LTS0029118 (magenta line), LTS0153650 (cyan line) and LTS0121786 (yellow line) complexed with **A)** *h*CA VA and **B)** LP. In the analysis Acetazolamide (AAZ) and Orlistat (ORL) were also included as red lines. The RMSD values reported were calculated on the ligand heavy atoms during MDs, superimposing the protein backbone. The reference structure was obtained from the first MD frame.

**FIGURE S4** **A-D)** Ligand atom interactions with the protein residues of *h*CA VA for compounds **A)** LTS0145253, **B)** LTS0029118, **C)** LTS0153650 and **D)** LTS0121786. **F-L)** Ligand atom interactions with the protein residues of LP for compounds **E)** LTS0145253, **F)** LTS0029118, **G)** LTS0153650 and **H)** LTS0121786. Only interactions that occur more than 30.0% of the simulation time in 200 ns of the trajectory are shown.

**FIGURE S5** RMSD trends for the LTS0059519 compound complexed with **A)** *h*CA VA and **B)** LP across three independent MD simulations. The RMSD values reported were calculated on the ligand heavy atoms during MDs, superimposing the protein backbone.

**FIGURE S6** Ligand atom interactions with the protein residues of *h*CA VA and LP, for the LTS0059519 compound complexed with **A-B)** *h*CA VA and **C-D)** LP across other two independent MD simulations. Only interactions that occur more than 30.0% of the simulation time in 200 ns of the trajectory are shown.

**FIGURE S7.** Pancreatic lipase % of inhibitory activity of orlistat (A) and LTS0059529 (B).

**FIGURE S8.** Secondary plots for the inhibition of LTS0059529 towards PL.

**FIGURE S9:** Receiver Operating Characteristic (ROC) curve for the enrichment study obtained using the Glide SP protocol (blue line) compared to a random model (gray line). The ROC curve demonstrates the trade-off between sensitivity (true positive rate) and Specificity (false positive rate).

**Table S1**. BindingDB code and canonical SMILES chemical formula of the active compounds against the PL.

**
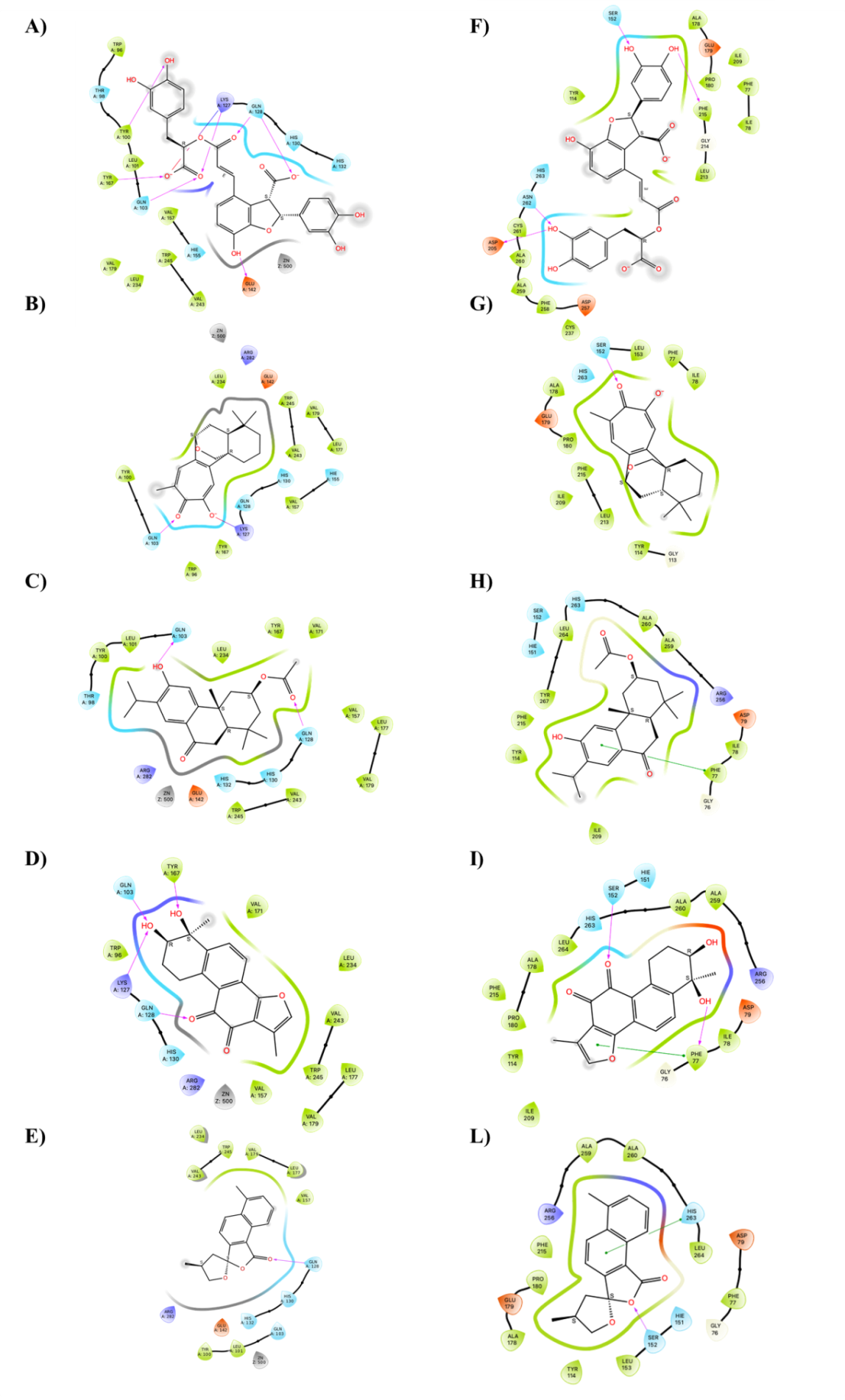
**

**FIGURE S1** **A-E)** 2D representation of *h*CA VA complexed with **A)** LTS0059529, **B)** LTS0145253, **C)** LTS0029118, **D)** LTS0153650, **E)** LTS0121786. **F-L)** 2D representation of PL complexed with **F)** LTS0059529, **G)** LTS0145253, **H)** LTS0029118, **I)** LTS0153650, **L)** LTS0121786. H-bonds, salt bridges and stacking interactions are shown as magenta, red-blue and green lines, respectively.


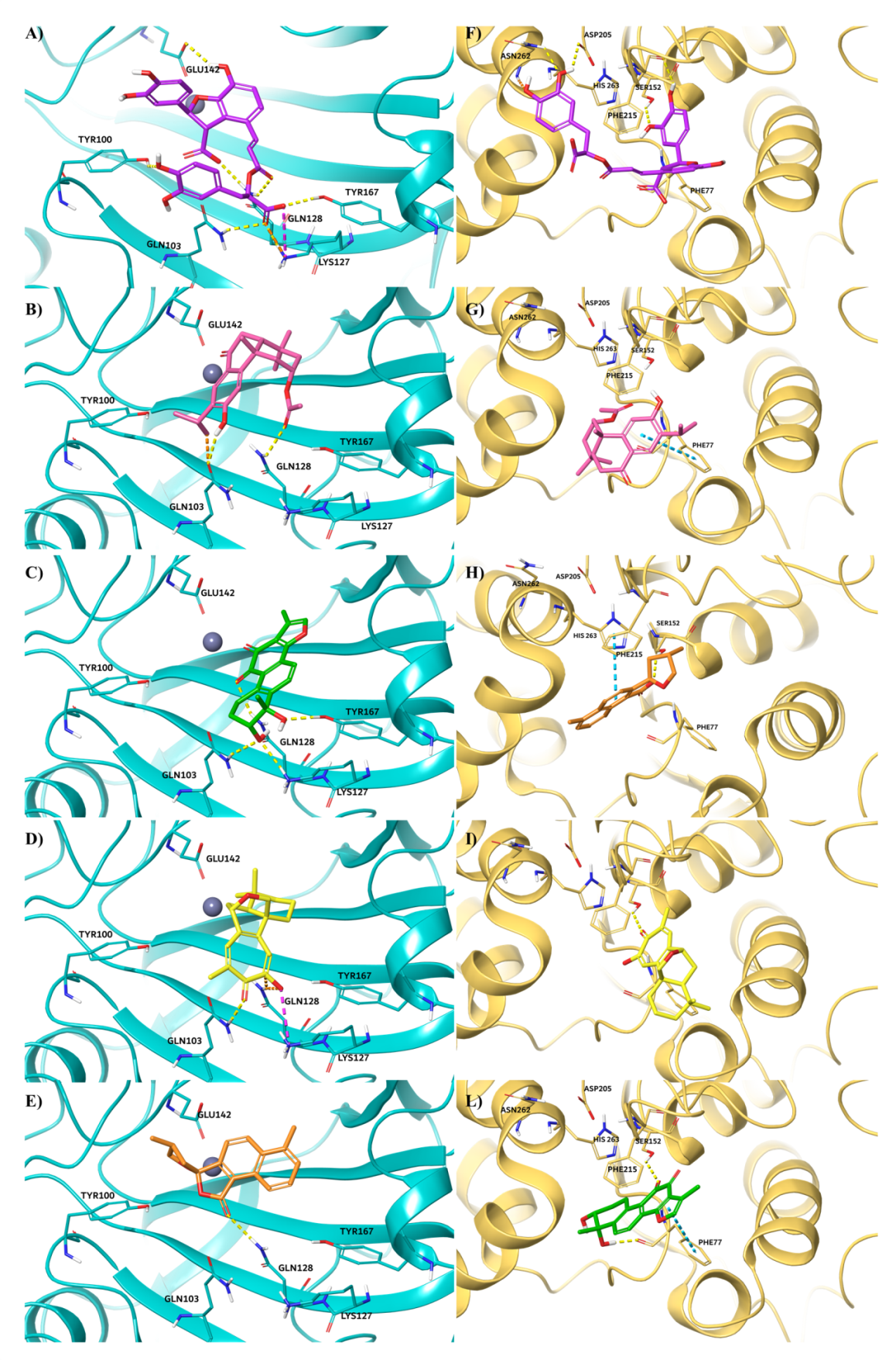


**FIGURE S2 A-E)** Three-dimensional representation of *h*CA VA complexed with **A)** LTS0059529, **B)** LTS0145253, **C)** LTS0029118, **D)** LTS0153650, **E)** LTS0121786. **F-L)** Three-dimensional representation of PL complexed with **F)** LTS0059529, **G)** LTS0145253, **H)** LTS0029118, **I)** LTS0153650, **L)** LTS0121786. *h*CA VA and PL are depicted as cyan and yellow cartoons, respectively. Ligands are depicted as sticks, while the amino acid residues involved in the most relevant contacts with ligands as cyan and yellow sticks. The zinc ion of the *h*CA VA is depicted as a grey sphere. H-bonds, salt bridges and stacking interactions are shown as yellow, magenta and cyan dash lines, respectively.

# **Analysis of docking binding poses**

The benzofuran-3-carboxylic acid component of the compound LTS0059529 forms crucial electrostatic interactions with the zinc ion, while the 1-carboxy-2-(3,4-dihydroxyphenyl)ethoxyl segment establishes four H-bonds with the side chains of TYR 100, GLN 103, LYS 127, and TYR 167. The carboxyl groups of the compound also form a salt bridge with LYS 127, and the ester carbonyl group accepts an H-bond from GLN 128. Two more H-bonds were observed between residues GLN128 and GLU 142 with the phenolic and carboxyl groups of the benzofuran ring, respectively (FIGURE S1-2A). When combined with the PL, the dihydroxyphenyl moiety linked to the benzofuran ring was involved in an H-bond acceptor with the side chain of the SER 152 and an H-bond donor with the backbone of PHE 215. Additionally, the second dihydroxyphenyl portion established an H-bond acceptor with the side chain of the ASN 262 and an H-bond donor with the ASP 205 (FIGURE S1-2F). The compound LTS0145253 did not show any interactions with the zinc ion in the *h*CA VA binding site. However, it engaged in an H-bond acceptors with GLN 103 and a salt bridge with the side chain of LYS 127, through its tropolon moiety. Hydrophobic interactions were observed with LEU 177, VAL 179, LEU 234, VAL 243 and TRP 245 residues (FIGURE S1-2B). On the other hand, in the PL complex, only an H-bond acceptor was established between the tropolon portion and the side chain of SER 152 (FIGURE S1-2G). The compound LTS0029118 in complex with *h*CA VA established an H-Bond donor between its phenolic group and the GLN 103 residue, an H-bond acceptor between the esterasic portion and the GLN 128, and an electrostatic interaction between the carbonyl group and the zinc ion (FIGURE S1-2C). As previously described, the compound LTS0029118 in complex with the PL interacts only through hydrophobic and a π-π interaction with the side chain of PHE 77 (FIGURE S1-2H). The compound LTS0153650 exhibited a good binding mode within the *h*AC VA binding site, with its two carbonyl groups positioned strategically towards the zinc ion. This arrangement facilitates the formation of highly favorable electrostatic interactions, and the compound's H-bond acceptor with GLN 128 further enhances its affinity. In addition, the diol portion established three H-bonds acceptor with LYS 127, GLN 103, and TYR 167 (FIGURE S1-2D). On the other hand, in the PL binding site, the compound LTS0153650 formed an H-bond donor and a π-π interaction with the backbone and the side chain of the residue PHE 77, respectively. Moreover, an H-bond acceptor was also observed between a carbonyl group of the compound LTS0153650 and the side chain of SER 152 (FIGURE S1-2I). Finally, in the binding site of the *h*CA VA, the compound LTS0121786 engaged an H-bond between its carbonyl group and the GLN 128 and several hydrophobic interactions with VAL 243, TRP 245, VAL 179, LEU 177, and VAL 157 through its aromatic portion (FIGURE S1-2E). Conversely, it interacted with the HIS 263 and the SER 152 residues of the PL binding site through a π-π interaction and an H-bond, respectively (FIGURE S1-2L).

**
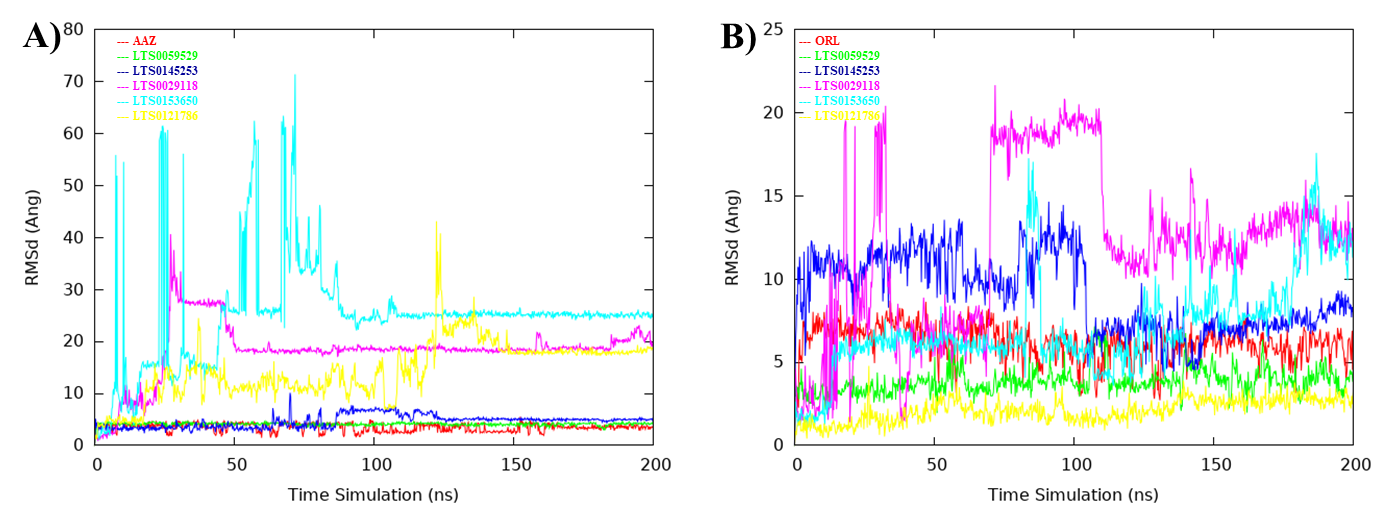
**

**FIGURE S3** RMSD trends of LTS0059519 (green line), LTS0145253 (blue line), LTS0029118 (magenta line), LTS0153650 (cyan line) and LTS0121786 (yellow line) complexed with **A)** *h*CA VA and **B)** LP. In the analysis Acetazolamide (AAZ) and Orlistat (ORL) were also included as red lines. The RMSD values reported were calculated on the ligand heavy atoms during MDs, superimposing the protein backbone. The reference structure was obtained from the first MD frame.


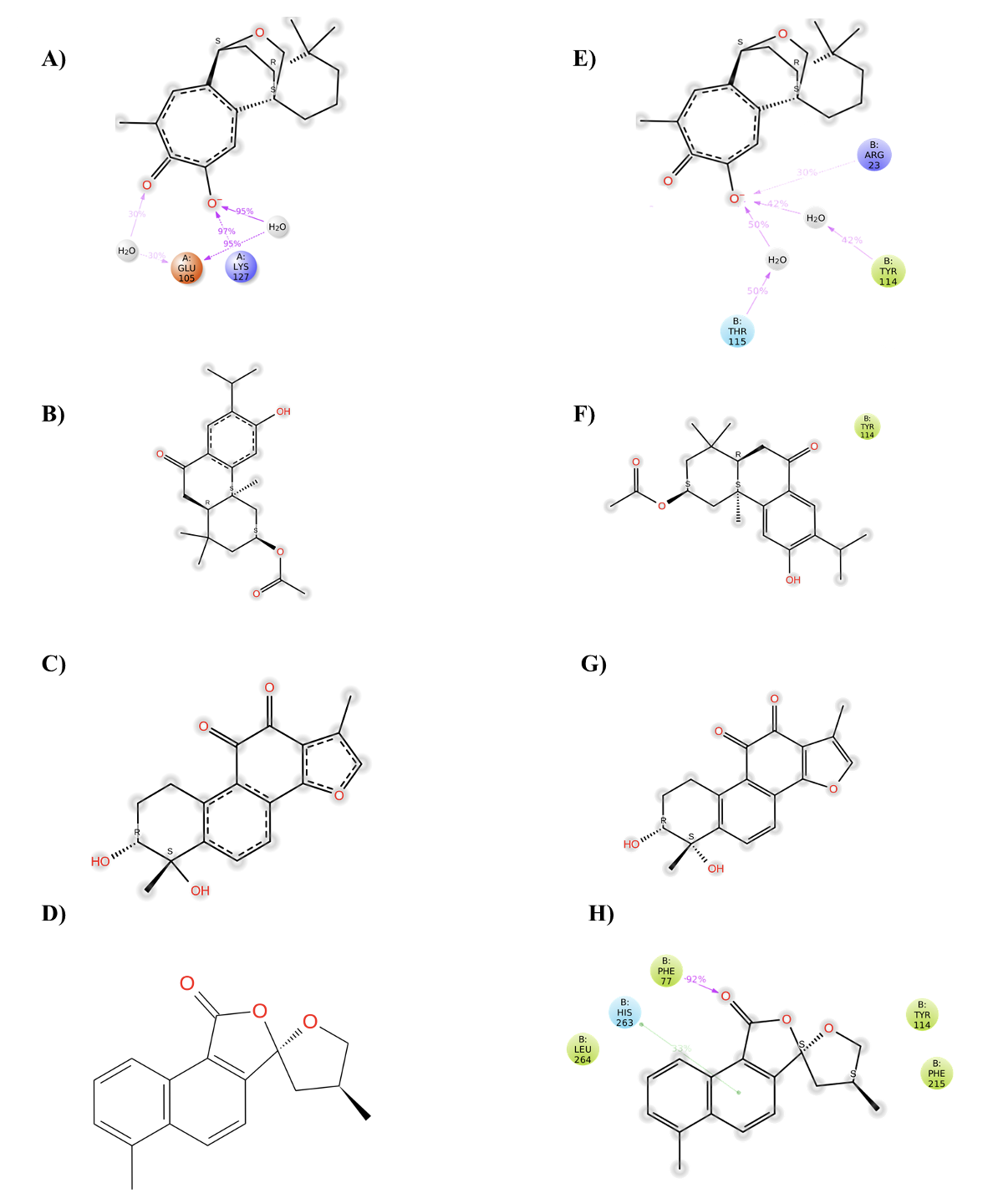


**FIGURE S4** **A-D)** Ligand atom interactions with the protein residues of *h*CA VA for compounds **A)** LTS0145253, **B)** LTS0029118, **C)** LTS0153650 and **D)** LTS0121786. **F-L)** Ligand atom interactions with the protein residues of LP for compounds **E)** LTS0145253, **F)** LTS0029118, **G)** LTS0153650 and **H)** LTS0121786. Only interactions that occur more than 30.0% of the simulation time in 200 ns of the trajectory are shown.

**
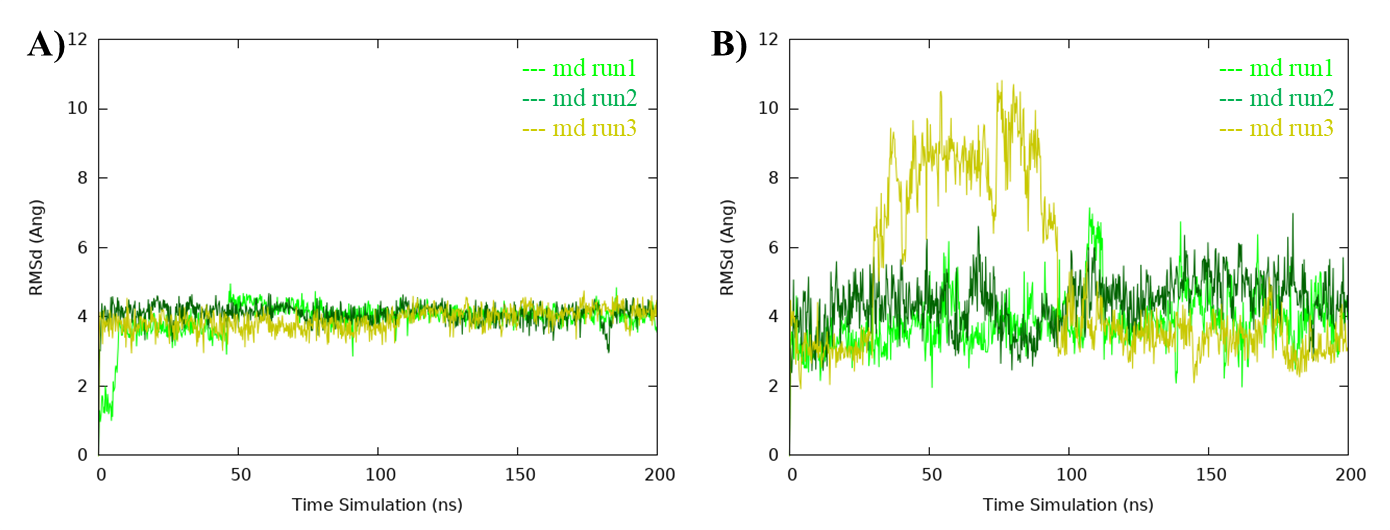
**

**FIGURE S5** RMSD trends for the LTS0059519 compound complexed with **A)** *h*CA VA and **B)** LP across three independent MD simulations. The RMSD values reported were calculated on the ligand heavy atoms during MDs, superimposing the protein backbone.


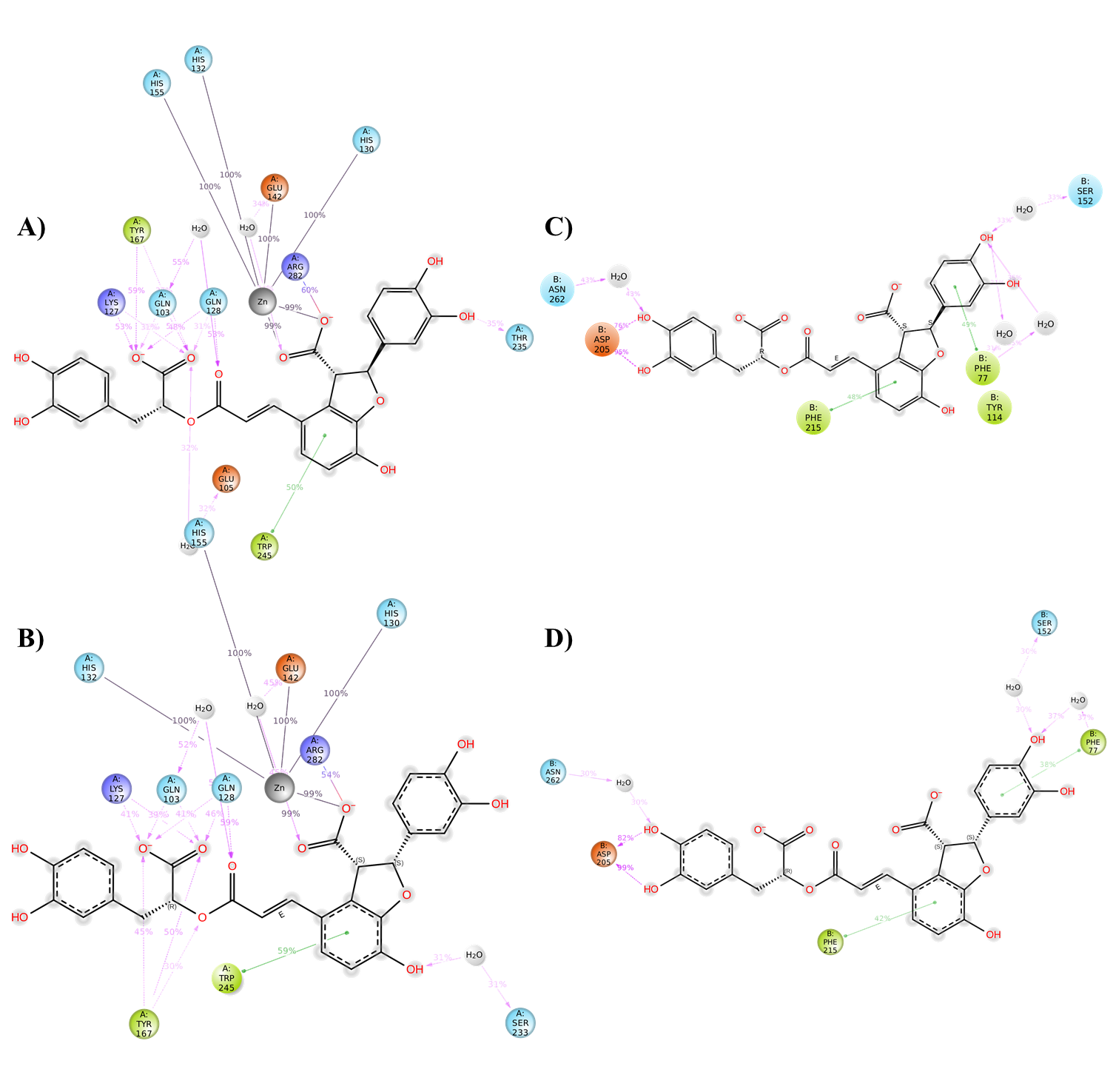


**FIGURE S6** Ligand atom interactions with the protein residues of *h*CA VA and LP, for the LTS0059519 compound complexed with **A-B)** *h*CA VA and **C-D)** LP across other two independent MD simulations. Only interactions that occur more than 30.0% of the simulation time in 200 ns of the trajectory are shown.


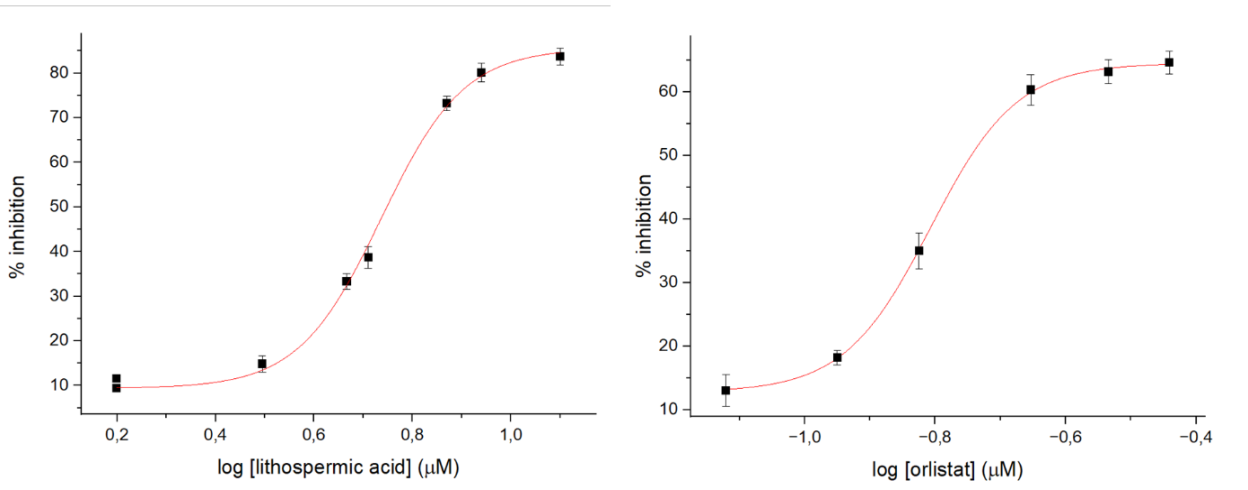


**FIGURE S7.** Pancreatic lipase % of inhibitory activity of orlistat (A) and LTS0059529 (B).


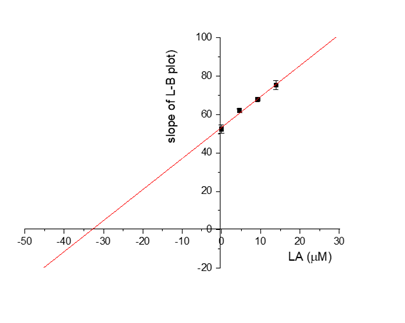


**FIGURE S8.** Secondary plots for the inhibition of LTS0059529 towards PL.


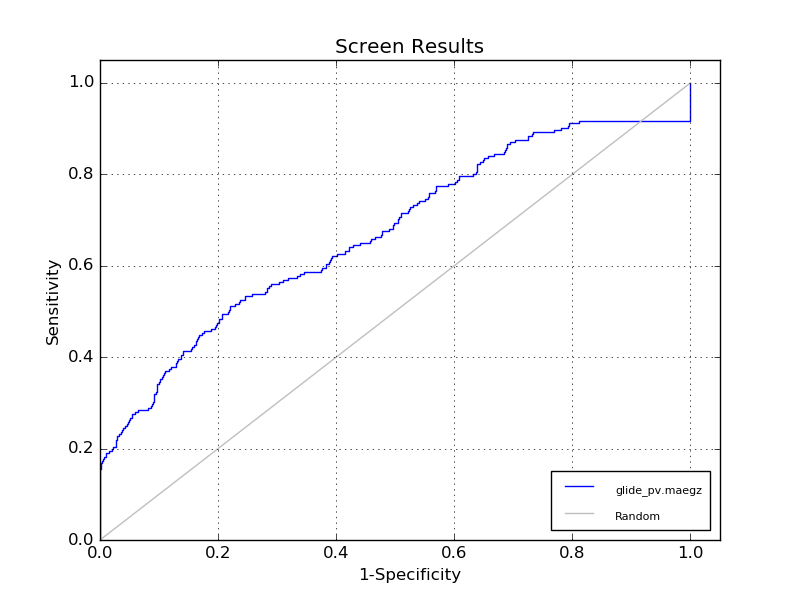


**FIGURE S9:** Receiver Operating Characteristic (ROC) curve for the enrichment study obtained using the Glide SP protocol (blue line) compared to a random model (gray line). The ROC curve demonstrates the trade-off between sensitivity (true positive rate) and Specificity (false positive rate).

**Table S1**. BindingDB code and canonical SMILES chemical formula of the active compounds against the PL.

| BindingDB | SMILES chemical formula |
| --- | --- |
| Morin | Oc1cc(O)cc(c12)oc(c(c2=O)O)-c3c(O)cc(O)cc3 |
| BindingDB_50401727_3D | Oc1cc(O)cc(c12)occc2=O |
| Gen | Oc1cc(O)cc(c12)occ(c2=O)-c3ccc(O)cc3 |
| BindingDB_50401723_3D | CC(C)=CCc(c1O)c(O)cc(c12)occ(c2=O)-c3ccc(O)cc3 |
| BindingDB_50401724_3D | c1cc(O)c(OC)cc1-c(c2=O)coc(c23)cc(O)cc3O |
| Biochanin | COc(cc1)ccc1-c(c2=O)coc(c23)cc(O)cc3O |
| BindingDB_51366121_3D | c1cccc(F)c1/C=C(/C2=O)Cc(c23)ccc(c3)O |
| BindingDB_51366118_3D | c1cccc(Cl)c1/C=C(/C2=O)Cc(c23)ccc(c3)O |
| BindingDB_51366113_3D | c1cc(O)ccc1/C=C(/C2=O)Cc(c23)ccc(c3)O |
| Naringenin | Oc1cc(O)cc(c12)O[C@@H](CC2=O)c3ccc(O)cc3 |
| BindingDB_50401719_3D | Oc1c(O)c(O)cc(c12)O[C@@H]([C@H](C2=O)O)c3ccc(O)cc3 |
| BindingDB_50381000_mol_1 | COc(cc1)c(O)cc1-c(c(c2=O)O)oc(c23)cc(O)cc3 |
| BindingDB_50380999_mol_1 | COc(cc1)ccc1-c(c(c2=O)O)oc(c23)cc(O)cc3 |
| BindingDB_50380998_mol_1 | COc1c(O)cc(cc1)[C@@H](CC2)Oc(c23)cc(O)cc3 |
| BindingDB_50240348_mol_1 | Oc1cc(O)cc(c12)O[C@@H]([C@H](C2=O)O)c3ccc(O)cc3 |
| BindingDB_51282461_3D | Oc1c(O)ccc(c1)[C@H]([C@H](C2)O)Oc(c23)c(c(O)cc3O)Cc(c4O)c(O)cc(c45)O[C@@H]([C@H](C5)O)c(c6)ccc(O)c6O |
| BindingDB_51282464_3D | Oc1c(O)ccc(c1)[C@H]([C@H](C2)O)Oc(c23)c(c(O)cc3O)Cc(c(O)cc4O)c(c45)O[C@@H]([C@H](C5)O)c(c6)ccc(O)c6O |
| BindingDB_50213686_mol_1 | [C@@H]12[C@@H]3[C@@H](O3)[C@@](O)(C(=O)[C@@H]1O2)C[C@@H](C(=O)OC)NC(=O)/C=C/CCCC/C=C/C |
| BindingDB_50542118_3D | C1=CC(C)(C)Oc(c(C)c2)c1c3[nH]c(c4c23)ccc(c4)OC |
| BindingDB_50346601_mol_1 | CC(C)(C1)CC[C@](C(=O)O)(CC2)[C@@H]1C([C@@]23C)=CC[C@H]4[C@@]3(C)CC[C@@H]5[C@]4(C)CC[C@H](O)C5(C)C |
| BindingDB_50337360_mol_1 | O=C1C[C@@H]([C@@H](O1)C)c2cc(OC)c(O)cc2 |
| BindingDB_50392475_mol_1 | c1cc(O)c(O)cc1\C=C\C(=O)O[C@@H](C(=O)O)[C@@H](O)COC(=O)c2cc(O)c(O)c(c2)O |
| BindingDB_50067040_mol_1 | c1cc(O)c(OC)cc1\C=C\C(=O)CC(=O)/C=C/c2cc(OC)c(O)cc2 |
| BindingDB_50337364_mol_1 | O=C(O)c1cc(OC)c(O)cc1 |
| BindingDB_50851656_3D | c1cc(O)ccc1CCC(=O)c2c(O)c(c(O)cc2O[C@H](O3)[C@H](O)[C@@H](O)[C@H](O)[C@H]3CO)Cc(c(O)c4C(=O)CCc5ccc(O)cc5)c(O)cc4O[C@H](O6)[C@H](O)[C@@H](O)[C@H](O)[C@H]6CO |
| BindingDB_50213694_mol_1 | [C@H]12[C@H]3[C@H](O3)[C@@](O)(C(=O)[C@H]1O2)C[C@H](C(=O)OC)NC(=O)/C=C/CCCC/C=C/C |
| BindingDB_50392474_mol_1 | O[C@@H]1CO[C@H]([C@H](O)[C@H]1O)O[C@H]([C@@H](O)[C@@H](O)[C@H]2CO)[C@@H](O2)Oc(c3O)c(-c4cc(O)c(O)cc4)oc(c35)cc(O)cc5=O |
| 1 | c1cc(O)ccc1CCC(=O)c2c(cc(O)cc2O)O[C@H](O3)[C@H](O)[C@@H](O)[C@H](O)[C@H]3CO |
| **2** | Oc1cc(O)cc(c12)O[C@@H]([C@H](C2)O)c(c3)ccc(O)c3O |
| BindingDB_50542117_3D | C1=CC(C)(C)Oc(c(C)c2)c1c3[nH]c(c4c23)cc(OC)c(c4)OC |
| BindingDB_50135527_mol_1 | C=C(C)[C@@H](C1)Oc(c12)ccc3c2O[C@H]4[C@@H](C3=O)c5c(OC4)cc(OC)c(c5)OC |
| BindingDB_4375_mol_1 | O=C(O)/C=C/c1cc(O)c(O)cc1 |
| BindingDB_50392477_mol_1 | O=C(O)[C@H](O)[C@@H](O)CO |
| BindingDB_50085536_mol_1 | Oc1cc(C(=O)O)cc(O)c1O |
| BindingDB_51277070_3D | NS(=O)(=O)NCc(o1)nnc1[C@H](S(=O)(=O)C)c(n2)sc(c23)cc(cc3)-c4ccccc4 |
| BindingDB_51366122_3D | c1cc(O)c(OC)cc1/C=C(/C2=O)Cc(c23)ccc(c3)OC |
| BindingDB_50542104_3D | COc1c(OC)ccc(c1)NC(=O)C(=O)c(c2)ccc3[nH]c(c4c23)cccc4 |
| BindingDB_51282462_3D | Oc1c(O)ccc(c1)[C@H]([C@H](C2)O)Oc(c23)c(c(O)cc3O)Cc(c4O)c(O)c(c(c45)O[C@@H]([C@H](C5)O)c(c6)ccc(O)c6O)Cc(c(O)cc7O)c(c78)O[C@@H]([C@H](C8)O)c(c9)ccc(O)c9O |
| BindingDB_50138737_mol_1 | c1ccccc1N(C)C(=O)n(n2)c(SCC(F)(F)F)nc2-c3ccc(Cl)cc3 |
| BindingDB_50138751_mol_1 | c1ccccc1N(C)C(=O)n(n2)c(SC)nc2-c3ccc(cc3)OC(F)(F)F |
| BindingDB_50138735_mol_1 | CC(C)c1c(C)n(oc1=O)C(=O)N(C2)CCC[C@@H]2C |
| BindingDB_429816_3D | c1cc(O)c(OC)cc1CC[C@@H](O)C[C@H](O)CCc2cc(OC)c(O)cc2 |
| BindingDB_429815_3D | c1cc(O)ccc1CC[C@@H](O)C[C@H](O)CCc2ccc(O)cc2 |
| BindingDB_50259746_mol_1 | c1cc(O)c(OC)cc1\C=C\C(=O)OCCc2ccc(O)cc2 |
| Untitled | c1c(O)cc(OC)c2OCCc(c3c12)cccc3 |
| Untitled | c1cccc(c1c23)CCOc2c(O)cc(c3)O |
| BindingDB_429805_3D | COc1cc(OC)cc2ccc(c3c12)cc(c(c3)O)OC |
| BindingDB_429801_3D | c1ccccc1CCc2cc(OC)cc(c2)OC |
| BindingDB_429802_3D | c1cccc(O)c1CCc2cc(OC)c(c(c2)O)OC |
| Phloretin | Oc1cc(O)cc(O)c1C(=O)CCc2ccc(O)cc2 |
| BindingDB_50401712_3D | c1cc(O)ccc1[C@H](CC2=O)Oc(c23)c(CC=C(C)C)c(O)c(c3O)Cc4ccc(O)cc4 |
| BindingDB_50542107_3D | CN(C)c1ccc(cc1)NC(=O)C(=O)c(c2)ccc3n(CC)c(c4c23)cccc4 |
| BindingDB_50333303_3D | c1cc(O)cc(c12)O[C@@H](CC2=O)c3c(O)cc(O)cc3 |
| BindingDB_50333306_3D | c1cc(O)c(O)c(c12)oc(c2)-c3cc(O)cc(c3)O |
| BindingDB_50333310_3D | c1c(O)c(O)cc(c12)oc(c2)-c3cc(O)cc(c3)O |
| BindingDB_50442403_mol_1 | CC(C)=CCc(c1O)c(O)c(CC=C(C)C)c(c12)occ(c2=O)-c3ccc(O)cc3 |
| BindingDB_50337361_mol_1 | O=C1C[C@H]([C@@H](O1)C)c2cc(OC)cc(c2)O |
| BindingDB_50241243_mol_1 | OC[C@@H]1[C@@H](O)[C@H](O)[C@@H](O)[C@@H](O1)Oc(c2=O)c(-c3ccc(O)cc3)oc(c24)cc(O)cc4O |
| BindingDB_50241354_mol_1 | OC[C@@H]1[C@@H](O)[C@H](O)[C@@H](O)[C@@H](O1)Oc(c2=O)c(-c3cc(O)c(O)cc3)oc(c24)cc(O)cc4O |
| BindingDB_50333297_3D | OC[C@@H]1[C@@H](O)[C@H](O)[C@@H](O)[C@@H](O1)Oc(c2=O)c(-c3ccc(cc3)OC)oc(c24)cc(O)cc4O |
| BindingDB_50269605_mol_1 | Oc1cc(O)cc(c12)O[C@H](CC2=O)c3c(O)cc(O)cc3 |
| BindingDB_50303002_mol_1 | CC(C)=CCC/C(C)=C/Cc1c(cc(O)cc1O)-c(c2)oc(c23)cc(O)cc3 |
| BindingDB_50333319_3D | o1ccc(c2)c1cc(c23)oc(c3)-c4cc(O)cc(c4)O |
| BindingDB_50269559_mol_1 | Oc1cc(O)cc(c12)oc(cc2=O)-c3c(O)cc(O)cc3 |
| Isorhamnetin | c1cc(O)c(OC)cc1-c(c(c2=O)O)oc(c23)cc(O)cc3O |
| kaempferol | Oc1cc(O)cc(c12)oc(c(c2=O)O)-c3ccc(O)cc3 |
| quercetin | Oc1cc(O)cc(c12)oc(c(c2=O)O)-c3cc(O)c(O)cc3 |
| BindingDB_50108046_mol_1 | c1cc(O)cc(O)c1/C=C/c2cc(O)cc(c2)O |
| BindingDB_50250915_mol_1 | c1cc(O)cc(c12)oc(c2)-c3cc(O)cc(c3)O |
| BindingDB_50177405_mol_1 | O=Cc1cc(OC)c(O)cc1 |
| BindingDB_51366119_3D | c1cc(Cl)c(Cl)cc1/C=C(/C2=O)Cc(c23)ccc(c3)O |
| BindingDB_50380997_mol_1 | c1cc(O)ccc1CCCc2c(OC)cc(O)cc2 |
| BindingDB_51366117_3D | c1cc(Br)ccc1/C=C(/C2=O)Cc(c23)ccc(c3)O |
| BindingDB_50401716_3D | CC(C)=CCc(c(O)cc1O)c(c12)O[C@H](CC2=O)c3ccc(O)cc3 |
| BindingDB_51282460_3D | Oc1c(O)ccc(c1)[C@H]([C@H](C2)O)Oc(c23)cc(O)c(c3O)Cc(c4O)c(O)cc(c45)O[C@@H]([C@H](C5)O)c(c6)ccc(O)c6O |
| BindingDB_50401717_3D | Oc1cc(O)cc(c12)O[C@@H](CC2=O)c3cc(O)cc(c3)O |
| BindingDB_51366133_3D | C=CCCCCCCCCNc(c1)ccc(c12)CC(/C2=O)=C\c3cc(OC)c(O)cc3 |
| 5j | c1ccccc1C(=O)/C=C/c2ccccc2 |
| BindingDB_50401713_3D | CC(C)(O)CCc(c(O)cc1O)c(c12)O[C@H](CC2=O)c(c3)c(O)cc(c34)OC(C)(C)C=C4 |
| BindingDB_50241625_mol_1 | c1cc(O)cc(c12)O[C@@H](CC2)c3ccc(O)cc3 |
| BindingDB_50251012_mol_1 | c1cc(O)ccc1CCCc2c(O)cc(cc2)OC |
| BindingDB_50401728_3D | CC(O1)(C)C=Cc(c2O)c1cc(c23)occc3=O |
| BindingDB_50542102_3D | Cc1ccc(cc1)NC(=O)C(=O)c(c2)ccc3n(c(c4c23)cccc4)Cc5ccc(Cl)cc5 |
| BindingDB_50542103_3D | c1ccccc1NC(=O)C(=O)c(c2)ccc3n(c(c4c23)cccc4)Cc5ccc(Cl)cc5 |
| BindingDB_51098598_3D | O=CCC(\C=O)=C/C[C@@H]1C(=C)CC[C@H]([C@@]12C)C(C)(C)CCC2 |
| BindingDB_51134808_3D | CC(C)=CCCC(=C)[C@@H](O)Cc(c(O)c1C(=O)C(C)C)c(O)c(c1O)CC(=C2O)C(=O)C(=C(O)C2(C)C)C(=O)[C@H](C)CC |
| BindingDB_50851655_3D | c1cc(O)ccc1CCC(=O)c2c(O)c(c(O)cc2O[C@H](O3)[C@H](O)[C@@H](O)[C@H](O)[C@H]3CO)Cc(c(O)cc4O)c(O)c4C(=O)CCc5ccc(O)cc5 |
| BindingDB_50542113_3D | CN(C)c1ccc(cc1)NC(=O)C(=O)c(c2)ccc3n(c(c4c23)cccc4)Cc5ccc(Cl)cc5 |
| BindingDB_51366111_3D | c1c(O)ccc(c12)CC(/C2=O)=C\c3cc(OC)c(OC)c(c3)OC |
| BindingDB_51366116_3D | c1ccc(Br)cc1/C=C(/C2=O)Cc(c23)ccc(c3)O |
| BindingDB_50542124_3D | COc(cc1)ccc1NC(=O)C(=O)c(c2)ccc3n(c(c4c23)cccc4)Cc5ccc(Cl)cc5 |
| BindingDB_51366107_3D | c1cc(O)ccc1/C=C(/C2=O)Cc(c23)cccc3 |
| BindingDB_50890321_3D | O=C(S1)NC(=O)\C1=C\c2c(-c(cc3)ccc3C)nn(c2)-c4ccccc4 |
| BindingDB_50542106_3D | c1ccnc(c12)ccc(c2)NC(=O)C(=O)c(c3)ccc4n(CC)c(c5c34)cccc5 |
| BindingDB_50326047_mol_1 | O=C(O)[C@@H](CS)NC(=O)[C@@H]([C@@H](C)O)NC(=O)[C@@H](CCC(=O)N)NC(=O)CNC(=O)[C@H]1CCCN1C(=O)[C@H](Cc2cnc[nH]2)NC(=O)[C@@H]3CCCN3C(=O)[C@H](CCC(=O)N)NC(=O)[C@@H](N)CS |
| BindingDB_50542115_3D | COc1c(OC)ccc(c1)NC(=O)C(=O)c(c2)ccc3n(CC)c(c4c23)cccc4 |
| BindingDB_50890313_3D | O=C(S1)NC(=O)\C1=C\c2c(-c3ccc([N+]([O-])=O)cc3)nn(c2)-c4ccccc4 |
| BindingDB_50542119_3D | CC(C)=CCC[C@](C)(C=C1)Oc(c(C)c2)c1c3[nH]c(c4c23)cccc4 |
| BindingDB_50242015_mol_1 | c1cc(O)cc(O)c1-c(c(c2=O)CC=C(C)C)oc(c23)c(CC=C(C)C)c(O)cc3O |
| BindingDB_51282463_3D | Oc1c(O)ccc(c1)[C@H]([C@H](C2)O)Oc(c23)c(c(O)cc3O)Cc(c4O)c(O)c(c(c45)O[C@@H]([C@H](C5)O)c(c6)ccc(O)c6O)Cc(c(c78)O[C@@H]([C@H](C8)O)c(c9)ccc(O)c9O)c(O)c(c7O)Cc(c(O)cc1O)c(c12)O[C@@H]([C@H](C2)O)c(c1)ccc(O)c1O |
| BindingDB_50890324_3D | O=C(S1)NC(=O)\C1=C\c2c(-c3ccccc3)nn(c2)-c4ccccc4 |
| BindingDB_50542110_3D | COc(cc1)ccc1NC(=O)C(=O)c(c2)ccc3[nH]c(c4c23)cccc4 |
| BindingDB_50542129_3D | c1cnccc1NC(=O)C(=O)c(c2)ccc3n(c(c4c23)cccc4)Cc5ccc(Cl)cc5 |
| BindingDB_51366112_3D | c1c(O)ccc(c12)CC(/C2=O)=C\c3c(OC)cc(OC)cc3OC |
| BindingDB_50542112_3D | c1ccnc(c12)ccc(c2)NC(=O)C(=O)c(c3)ccc4n(c(c5c34)cccc5)Cc6ccc(Cl)cc6 |
| BindingDB_50542123_3D | COc1c(OC)cc(cc1OC)NC(=O)C(=O)c(c2)ccc3n(C)c(c4c23)cccc4 |
| BindingDB_50542120_3D | c1ccccc1NC(=O)C(=O)c(c2)ccc3n(CC)c(c4c23)cccc4 |
| BindingDB_51366110_3D | COc(cc1)cc(OC)c1/C=C(/C2=O)Cc(c23)ccc(c3)O |
| BindingDB_51366129_3D | C=CCCCCNc(c1)ccc(c12)CC(/C2=O)=C\c3cc(OC)c(O)cc3 |
| BindingDB_50392476_mol_1 | c1cc(O)c(O)cc1\C=C\C(=O)O[C@H]([C@@H](O)C(=O)O)COC(=O)c2cc(O)c(O)c(c2)O |
| BindingDB_50542116_3D | c1ccc(OC)cc1NC(=O)C(=O)c(c2)ccc3n(c(c4c23)cccc4)Cc5ccc(Cl)cc5 |
| BindingDB_50213689_mol_1 | [C@@H]12[C@@H]3[C@@H](O3)[C@](O)(C(=O)[C@@H]1O2)C[C@@H](C(=O)OC)NC(=O)/C=C/CCCC/C=C/C |
| BindingDB_50542114_3D | CC(C)=CCC/C(C)=C/CCC(C(=O)O1)=CC12c3c(ccc(c3)O)OC(=O)C2(CC(=O)c4c(O)ccc(c4)O)CC\C=C(C)\CCC=C(C)C |
| BindingDB_50429042_mol_1 | c1cc(F)ccc1NC(=O)C(=O)c(c2)ccc3n(c(c4c23)cccc4)Cc5ccc(Cl)cc5 |
| BindingDB_392707_3D | O[C@H](O1)[C@@](O2)(O)[C@H]2C[C@@]13c4c(c5c(cc4)O[C@H](C5)C(=C)C)O[C@H]6[C@@H]3c7c(OC6)cc(OC)c(c7)OC |
| BindingDB_50381001_mol_1 | O[C@@H](O1)[C@](O2)(O)[C@@H]2C[C@@]13c4c(c5c(cc4)O[C@H](C5)C(=C)C)O[C@H]6[C@@H]3c7c(OC6)cc(OC)c(c7)OC |
| BindingDB_51366115_3D | C1=CC(=O)C=C(OC)[C@]1(O)CCCc2ccc(O)cc2 |
| BindingDB_50333324_3D | c1cccc(Br)c1/C=C(/C2=O)Cc(c23)ccc(c3)O |
| BindingDB_50542108_3D | CC(O1)(C)C=Cc(c2)c1cc(c23)oc(c3)-c4cc(O)cc(c4)O |
| BindingDB_50251014_mol_1 | c1cnccc1NC(=O)C(=O)c(c2)ccc3[nH]c(c4c23)cccc4 |
| BindingDB_50542125_3D | CC(C)=CCc(c1)c(O)cc(c12)oc(c2)-c3cc(O)cc(c3)O |
| BindingDB_50381284_mol_1 | c1ccccc1NC(=O)C(=O)c(c2)ccc3[nH]c(c4c23)cccc4 |
| BindingDB_51366120_3D | CC(C)=CCc1c(O)cc(cc1O)-c(c2)oc(c23)cc(O)cc3 |
| BindingDB_50542111_3D | c1cc(F)ccc1/C=C(/C2=O)Cc(c23)ccc(c3)O |
| BindingDB_50542128_3D | Cc1cnc(s1)NC(=O)C(=O)c(c2)ccc3n(c(c4c23)cccc4)Cc5ccc(Cl)cc5 |
| BindingDB_51366114_3D | c1ccc(OC)cc1NC(=O)C(=O)c(c2)ccc3[nH]c(c4c23)cccc4 |
| BindingDB_50371232_mol_1 | c1c(O)ccc(c12)CC(/C2=O)=C\c3c(OC)cc(OC)c(c3)OC |
| BindingDB_50542127_3D | Oc1c(O)c(C(C)C)cc(c1[C@]23C(=O)O)CC[C@H]2C(C)(C)CCC3 |
| BindingDB_50686041_3D | c1ccccc1NC(=O)C(=O)c(c2)ccc3n(C)c(c4c23)cccc4 |
|  | CC(C)=CC[C@@]12[C@@H](OC1=O)CC(=C2)C(OC)OC |
| BindingDB_50576045_3D | c1cc(O)c(OC)cc1/C=C(/C2=O)Cc(c23)ccc(c3)N4CCCCC4 |
| BindingDB_51366126_3D | CCCCCC[C@H](C1=C)[C@@H](O1)C[C@@H](OC(=O)[C@@H](NC=O)CC(C)C)CCCCCCCCCCC |
| BindingDB_51409224_3D | c1cc(O)c(OC)cc1/C=C(/C2=O)Cc(c23)ccc(c3)N4CCCC4 |
| BindingDB_51098590_3D | C#CCOc(cc1)ccc1C(=O)O[C@H](C[C@H](O2)[C@@H](C2=O)CCCCCC)CCCCCCCCCCC |
| BindingDB_51409225_3D | C1CCC(C)(C)[C@@H]([C@]12C)CCC(=C)[C@H]2C\C=C(\C=O)CC(=O)OCc3cn(nn3)Cc4c(Br)ccc(c4)OC |
| BindingDB_50890318_3D | CCCCCC[C@H](C1=O)[C@@H](O1)C[C@@H](OC(=O)c2cc(OC)ccc2)CCCCCCCCCCC |
| BindingDB_50851652_3D | c1ccccc1-n(c2)nc(-c3ccc(Cl)cc3)c2/C=C4/C(=O)N(C(=O)S4)Cc5ccc([N+]([O-])=O)cc5 |
| BindingDB_51098585_3D | c1cc(O)ccc1CCC(=O)c2c(O)c(c(O)cc2O)Cc(c(O)cc3O)c(O)c3C(=O)CCc4ccc(O)cc4 |
| BindingDB_50890317_3D | C1CCC(C)(C)[C@@H]([C@]12C)CCC(=C)[C@H]2C\C=C(\C=O)CC(=O)OCc3cn(nn3)Cc4ccccc4 |
| BindingDB_51409227_3D | c1ccccc1-n(c2)nc(-c3ccc(F)cc3)c2/C=C4/C(=O)N(C(=O)S4)Cc5ccc([N+]([O-])=O)cc5 |
| BindingDB_50251013_mol_1 | CCCCCC[C@H](C1=O)[C@@H](O1)C[C@@H](OC(=O)c2ccc(cc2)OC)CCCCCCCCCCC |
| BindingDB_51098580_3D | CC(C)=CCc(c(O)cc1)c(O)c1C(=O)/C=C/c2c(O)cc(O)cc2 |
| BindingDB_392706_3D | C1CCC(C)(C)[C@@H]([C@]12C)CCC(=C)[C@H]2C\C=C(\C=O)CC(=O)OCc3cn(nn3)CC(=O)c4ccccc4 |
| BindingDB_50542131_3D | C=C(C)[C@@H](C1)Oc(cc2)c1c(c2[C@]34CO4)O[C@H]5[C@@H]3c6c(OC5)cc(OC)c(c6)OC |
| BindingDB_50193719_mol_1 | COc1c(OC)ccc(c1)NC(=O)C(=O)c(c2)ccc3n(c(c4c23)cccc4)Cc5ccc(Cl)cc5 |
| BindingDB_51366106_3D | CC(C)=CCc(c1O)c(O)cc(c12)O[C@@H](CC2=O)c(c3)c(O)cc(c34)OC(C)(C)C=C4 |
|  | c1cc(O)c(OC)cc1/C=C(/C2=O)Cc(c23)cccc3 |
| BindingDB_51134807_3D | C#CCOC(=O)CC(\C=O)=C/C[C@@H]1C(=C)CC[C@H]([C@@]12C)C(C)(C)CCC2 |
| BindingDB_50890319_3D | CC(C)=CCC/C(C)=C/Cc(c(O)c1C(=O)C(C)C)c(O)c(c1O)CC(=C2O)C(=O)C(=C(O)C2(C)C)C(=O)[C@H](C)CC |
| BindingDB_51366124_3D | c1ccccc1-n(c2)nc(-c3ccc([N+]([O-])=O)cc3)c2/C=C4/C(=O)N(C(=O)S4)Cc5ccc([N+]([O-])=O)cc5 |
| BindingDB_50542132_3D | c1cc(O)c(OC)cc1/C=C(/C2=O)Cc(c23)ccc(c3)OC(C)C |
| BindingDB_50193723_mol_1 | COc1c(OC)cc(cc1OC)NC(=O)C(=O)c(c2)ccc3n(c(c4c23)cccc4)Cc5ccc(Cl)cc5 |
| BindingDB_50686039_3D | CC(C)=CCc(c1O)c(O)cc(c12)O[C@@H](CC2=O)c(c3)c(O)cc(O)c3CC=C(C)C |
| BindingDB_50337363_mol_1 | OC/C(C)=C/C[C@@]12[C@@H](OC1=O)CC(=C2)CO |
| BindingDB_50890316_3D | C[C@H](O)[C@@H](O)c1cc(OC)c(O)cc1 |
| BindingDB_50542109_3D | c1ccccc1-n(c2)nc(-c3ccc(cc3)OC)c2/C=C4/C(=O)N(C(=O)S4)Cc5ccc([N+]([O-])=O)cc5 |
| BindingDB_50890315_3D | COc1c(OC)cc(cc1OC)NC(=O)C(=O)c(c2)ccc3[nH]c(c4c23)cccc4 |
|  | c1ccccc1-n(c2)nc(-c(cc3)ccc3C)c2/C=C4/C(=O)N(C(=O)S4)Cc5ccc([N+]([O-])=O)cc5 |
| BindingDB_50138734_mol_1 | CC(C)(C)c1ccc(cc1)C(=O)Nc2ccc(cc2)-n(c(=O)o3)nc3OC |
| BindingDB_51098581_3D | CCOc1nn(c(=O)o1)-c2cc(ccc2)OCc3ccccc3 |
| BindingDB_50890312_3D | O=C(O)CC(\C=O)=C/C[C@@H]1C(=C)CC[C@H]([C@@]12C)C(C)(C)CCC2 |
| BindingDB_51098600_3D | O=C(S1)NC(=O)\C1=C\c2c(-c3ccc(Cl)cc3)nn(c2)-c4ccccc4 |
| BindingDB_51366108_3D | C1CCC(C)(C)[C@@H]([C@]12C)CCC(=C)[C@H]2C\C=C(\C=O)CC(=O)OCc3cn(nn3)Cc4c(cccc4)-c5ccccc5 |
| BindingDB_50542126_3D | c1cc(O)c(OC)cc1/C=C(/C2=O)Cc(c23)ccc(c3)O |
| BindingDB_50337362_mol_1 | COc1c(OC)cc(cc1OC)NC(=O)C(=O)c(c2)ccc3n(CC)c(c4c23)cccc4 |
| BindingDB_51366109_3D | C[C@H](O)[C@H](O)c1cc(OC)c(O)cc1 |
| BindingDB_50890314_3D | c1cc(O)c(OC)cc1/C=C(/C2=O)Cc(c23)ccc(c3)N |
| BindingDB_50890323_3D | c1ccccc1-n(c2)nc(-c3ccccc3)c2/C=C4/C(=O)N(C(=O)S4)Cc5ccc([N+]([O-])=O)cc5 |
| BindingDB_50213683_mol_1 | O=C(S1)NC(=O)\C1=C\c2c(-c3ccc(F)cc3)nn(c2)-c4ccccc4 |
| BindingDB_50890322_3D | Oc1c(O)c(C(C)C)cc(c1[C@]234)[C@@H](OC2=O)C[C@H]3C(C)(C)CCC4 |
|  | O=C(S1)NC(=O)\C1=C\c2c(-c3ccc(cc3)OC)nn(c2)-c4ccccc4 |
| Orlistat | CCCCCCC(C1=O)C(O1)CC(OC(=O)C(NC=O)CC(C)C)C/C=C\C/C=C\CCCCC |
| BindingDB_51366134_3D | CCCCCC[C@H](C1=O)[C@@H](O1)C[C@@H](OC(=O)[C@@H](NC=O)CC(C)C)CCCCCCCCCCC |
| BindingDB_50494715_3D | c1cc(O)c(OC)cc1/C=C(/C2=O)Cc(c23)ccc(c3)N(CCCCCCCCCCCCO)CCCCCCCCCCCCO |
| BindingDB_50494713_3D | CC(C)=CCC/C(C)=C\CC/C(C)=C\Cc1c(c(CC=C(C)C)c(O)cc1O)-c(c2)oc(c23)cc(O)cc3 |
| BindingDB_50494714_3D | CC(C)=CCC/C(C)=C\Cc(c(O)cc1)c(c12)oc(c2)-c(cc3O)cc(c34)occ4 |
| BindingDB_51366132_3D | CC(C)=CCC/C(C)=C\CC/C(C)=C\Cc1c(cc(O)cc1OC)-c(c2)oc(c23)cc(O)cc3 |
| BindingDB_51366135_3D | C=CCCCCCCCCN(CCCCCCCCC=C)c(c1)ccc(c12)CC(/C2=O)=C\c3cc(OC)c(O)cc3 |
| BindingDB_50303004_mol_1 | c1cc(O)c(OC)cc1/C=C(/C2=O)Cc(c23)ccc(c3)NCCCCCCCCCCCCO |
| BindingDB_50494708_3D | CC(C)=CCC/C(C)=C\CC/C(C)=C\Cc1c(cc(O)cc1O)-c(c2)oc(c23)cc(O)cc3 |
| BindingDB_51098586_3D | CC(C)=CCC/C(C)=C\Cc(c1O)c(O)cc(c12)O[C@@H](CC2=O)c(ccc3O)c(O)c3CC=C(C)C |
| BindingDB_50494712_3D | C1CCC(C)(C)[C@@H]([C@]12C)CCC(=C)[C@H]2C\C=C(\C=O)CC(=O)OCc3cn(nn3)Cc4ccc(F)cc4 |
| BindingDB_51098582_3D | CC(C)=CCC[C@](C)(C=C1)Oc(c12)cc(cc2O)-c(c3)oc(c34)cc(O)cc4 |
| BindingDB_51098577_3D | C1CCC(C)(C)[C@@H]([C@]12C)CCC(=C)[C@H]2C\C=C(\C=O)CC(=O)OCc3cn(nn3)Cc4ccc(Cl)cc4 |
| BindingDB_51366130_3D | C1CCC(C)(C)[C@@H]([C@]12C)CCC(=C)[C@H]2C\C=C(\C=O)CC(=O)OCc3cn(nn3)Cc4ccc(cc4)SC |
| BindingDB_51098589_3D | c1cc(O)c(OC)cc1/C=C(/C2=O)Cc(c23)ccc(c3)N(CC[C@H](C)CCCC(C)C)CC[C@H](C)CCCC(C)C |
| BindingDB_50494707_3D | C1CCC(C)(C)[C@@H]([C@]12C)CCC(=C)[C@H]2C\C=C(\C=O)CC(=O)OCc3cn(nn3)Cc4ccc(cc4)OC |
| BindingDB_51098587_3D | CC(C)=CCC/C(C)=C\Cc(c(OC)cc(c1)O)c1/C=C/c2ccc(O)cc2 |
| BindingDB_51098588_3D | C1CCC(C)(C)[C@@H]([C@]12C)CCC(=C)[C@H]2C\C=C(\C=O)CC(=O)OCc3cn(nn3)Cc4ccc([N+]([O-])=O)cc4 |
| BindingDB_50494711_3D | C1CCC(C)(C)[C@@H]([C@]12C)CCC(=C)[C@H]2C\C=C(\C=O)CC(=O)OCc3cn(nn3)Cc(cc4)ccc4C(C)(C)C |
| BindingDB_51134709_3D | CC(C)=CCC/C(C)=C\Cc(c(O)cc1)c(c12)oc(c2)-c(c3CC=C(C)C)cc(O)cc3OC |
| BindingDB_51098599_3D | CCCCCC[C@@H](C1=O)[C@H](O1)C[C@@H](OC(=O)[C@H](NC=O)CC(C)C)CCCCCCCCCCC |
| BindingDB_51366127_3D | C1CCC(C)(C)[C@@H]([C@]12C)CCC(=C)[C@H]2C\C=C(\C=O)CC(=O)OCc3cn(nn3)Cc4c(Br)cc(F)cc4 |
| BindingDB_51366128_3D | c1cc(O)c(OC)cc1/C=C(/C2=O)Cc(c23)ccc(c3)N(CC)CC |
| BindingDB_51409226_3D | C=CCCCCN(CCCCC=C)c(c1)ccc(c12)CC(/C2=O)=C\c3cc(OC)c(O)cc3 |
| BindingDB_51366131_3D | CCCCCC[C@H](C1=O)[C@@H](O1)C[C@@H](OC(=O)c2ccc(O)cc2)CCCCCCCCCCC |
| BindingDB_51366123_3D | c1cc(O)c(OC)cc1/C=C(/C2=O)Cc(c23)ccc(c3)NCC[C@H](C)CCCC(C)C |
| Untitled_Molecule_000236 | c1cc(O)c(OC)cc1/C=C(/C2=O)Cc(c23)ccc(c3)OCCC |
| BindingDB_50559541_3D | c1cc(Br)ccc1Cc(nc(c23)cccc3)n(c2=O)CC(=O)NNC(=O)c(c4)c(=O)oc(c45)cccc5 |
| BindingDB_50559536_3D | CCCC(=O)OCC(OC(=O)CCC)COC(=O)CCC |
| BindingDB_429796_3D | CCCC(=O)O[C@@H]1C[C@@H]([C@@H]12)C=CC2 |
| BindingDB_429813_3D | c1ccc(O)c(OC)c1CCc2cc(O)cc(c2)O |
| BindingDB_429814_3D | c1cc(O)ccc1CCC(=O)/C=C/C=C/c2ccc(O)cc2 |
| BindingDB_429804_3D | c1cc(O)ccc1CCC(=O)/C=C/C=C/c2cc(OC)c(O)cc2 |
| BindingDB_429794_3D | COc1cc(O)cc2ccc(c3c12)cc(O)c(c3)OC |
| BindingDB_429812_3D | c1c(O)cc(O)cc1CCc2cc(OC)c(O)cc2 |
| Untitled | c1cc(O)ccc1\C=C\C(=O)/C=C/C=C/c2ccc(O)cc2 |
| BindingDB_429799_3D | Oc1cc(OC)cc2CCc(c3c12)cc(O)cc3 |
| BindingDB_50211956_mol_1 | COc(c(c1)O)c(O)cc1CCc2ccccc2 |
| BindingDB_429795_3D | c1cccc(O)c1CCc2cc(OC)cc(c2)O |
| BindingDB_429797_3D | c1cccc(O)c1CCc2cc(O)cc(c2)O |
| BindingDB_429810_3D | c1c(O)cc(OC)cc1CCc2cc(O)ccc2 |
| 442705 | c1cc(O)ccc1\C=C\C(=O)OCCc2ccc(O)cc2 |
| BindingDB_50381859_mol_1 | COc1cc(O)cc2CCc(c3c12)cccc3O |
| BindingDB_429800_3D | c1cccc(c12)ccc(c2)[C@@H](C(=O)N)NC(=O)[C@H](c(c3)ccc(c34)cccc4)NC(=O)CCC(=O)Nc5ccc(cc5)OCCCC |
| BindingDB_429803_3D | c1cccc(O)c1CCc2cc(OC)cc(c2)OC |
| BindingDB_50381861_mol_1 | c1cccc(O)c1CCc2cc(OC)c(OC)c(c2)OC |
| BindingDB_50381860_mol_1 | c1ccccc1C[C@@H](C(=O)N)NC(=O)[C@H](Cc2ccccc2)NC(=O)CCC(=O)Nc3ccc(cc3)OCCCCCCC |
| BindingDB_50381858_mol_1 | c1ccccc1C[C@@H](C(=O)N)NC(=O)[C@H](Cc2ccc(O)cc2)NC(=O)CCC(=O)Nc3ccc(cc3)OCCCC |
| BindingDB_51134702_3D | c1cc(O)ccc1C[C@@H](C(=O)N)NC(=O)[C@H](Cc2ccccc2)NC(=O)CCC(=O)Nc3ccc(cc3)OCCCC |
| BindingDB_51134706_3D | CCCCCC[C@H](C1=O)[C@@H](O1)C[C@H](OC(=O)[C@H](NC=O)CC(C)C)CCCCCCCCCCC |
| BindingDB_51134705_3D | CCCCCC[C@H](C1=O)[C@H](O1)C[C@@H](OC(=O)[C@@H](NC=O)CC(C)C)CCCCCCCCCCC |
| BindingDB_50274723_mol_1 | CCCCCC[C@@H](C1=O)[C@H](O1)C[C@@H](OC(=O)[C@@H](NC=O)CC(C)C)CCCCCCCCCCC |
| BindingDB_51134701_3D | CCCCCC[C@H](C1=O)[C@@H](O1)C[C@@H](OC(=O)[C@H](NC=O)CC(C)C)CCCCCCCCCCC |
| BindingDB_51134710_3D | CCCCCC[C@H](C1=O)[C@@H](O1)C[C@H](OC(=O)[C@@H](NC=O)CC(C)C)CCCCCCCCCCC |
| BindingDB_51134704_3D | CCCCCC[C@@H](C1=O)[C@H](O1)C[C@H](OC(=O)[C@@H](NC=O)CC(C)C)CCCCCCCCCCC |
| BindingDB_50303006_mol_1 | CCCCCC[C@@H](C1=O)[C@H](O1)C[C@H](OC(=O)[C@H](NC=O)CC(C)C)CCCCCCCCCCC |
|  | CC(C)=CCC/C(C)=C/Cc(c(O)cc1)c(c12)oc(c2)-c(c3CC=C(C)C)cc(O)cc3O |
